# Increased PARylation impacts the DNA methylation process in type 2 diabetes mellitus

**DOI:** 10.1186/s13148-021-01099-1

**Published:** 2021-05-17

**Authors:** Michele Zampieri, Maria Giulia Bacalini, Ilaria Barchetta, Stefania Scalea, Flavia Agata Cimini, Laura Bertoccini, Stefano Tagliatesta, Giovanna De Matteis, Giuseppe Zardo, Maria Gisella Cavallo, Anna Reale

**Affiliations:** 1 Department of Experimental Medicine, Faculty of Medicine and Dentistry, Sapienza University of Rome, 00161 Rome, Italy; 2 IRCCS Istituto delle Scienze Neurologiche di Bologna, 40139 Bologna, Italy; 3 Research Centre for Animal Production and Aquaculture, Consiglio Per La Ricerca in Agricoltura E L’Analisi Dell’Economia Agraria (CREA), 00015 Monterotondo, Italy

**Keywords:** Epigenetics, PARylation, DNA methylation, Type 2 diabetes mellitus, 5-Hydroxymethylcytosine

## Abstract

**Background:**

Epigenetic modifications, such as DNA methylation, can influence the genetic susceptibility to type 2 diabetes mellitus (T2DM) and the progression of the disease. Our previous studies demonstrated that the regulation of the DNA methylation pattern involves the poly(ADP-ribosyl)ation (PARylation) process, a post-translational modification of proteins catalysed by the poly(ADP-ribose) polymerase (PARP) enzymes. Experimental data showed that the hyperactivation of PARylation is associated with impaired glucose metabolism and the development of T2DM. Aims of this case–control study were to investigate the association between PARylation and global and site-specific DNA methylation in T2DM and to evaluate metabolic correlates.

**Results:**

Data were collected from 61 subjects affected by T2DM and 48 healthy individuals, recruited as controls. Global levels of poly(ADP-ribose) (PAR, a surrogate of PARP activity), cytosine methylation (5-methylcytosine, 5mC) and de-methylation intermediates 5-hydroxymethylcytosine (5hmC) and 5-formylcytosine (5fC) were determined in peripheral blood cells by ELISA-based methodologies. Site-specific DNA methylation profiling of *SOCS3*, *SREBF1* and *TXNIP* candidate genes was performed by mass spectrometry-based bisulfite sequencing, methyl-sensitive endonucleases digestion and by DNA immuno-precipitation. T2DM subjects presented higher PAR levels than controls. In T2DM individuals, increased PAR levels were significantly associated with higher HbA1c levels and the accumulation of the de-methylation intermediates 5hmC and 5fC in the genome. In addition, T2DM patients with higher PAR levels showed reduced methylation with increased 5hmC and 5fC levels in specific *SOCS3* sites, up-regulated *SOCS3* expression compared to both T2DM subjects with low PAR levels and controls.

**Conclusions:**

This study demonstrates the activation of PARylation processes in patients with T2DM, particularly in those with poor glycaemic control. PARylation is linked to dysregulation of DNA methylation pattern via activation of the DNA de-methylation cascade and may be at the basis of the differential gene expression observed in presence of diabetes.

**Supplementary Information:**

The online version contains supplementary material available at 10.1186/s13148-021-01099-1.

## Background

Type 2 diabetes mellitus (T2DM) is an expanding global health problem and the rate of increase shows no signs of slowing [1]. Lifestyle and environmental factors such as diet, lack of exercise and age, contribute to the aetiology of T2DM. Because epigenetic modifications represent important links between genetic and environmental cues, altered epigenetic marks have been proposed to increase susceptibility to the disease and to underpin its development by impinging the expression of multiple genes. Epigenetic modifications refer to changes in gene function that do not result from altered DNA sequence, but foresee changes in chromatin structure [2]. DNA methylation—the addition of a methyl group to cytosine, mostly in the context of CpG dinucleotides [3]—is the most studied epigenetic mechanism. DNA methylation plays an important role in the development of several diseases, such as T2DM itself [4–7]. This is because of its potential to alter gene expression directly, by inhibiting the binding of specific transcription factors, and indirectly, by recruiting methyl-CpG-binding proteins and their associated repressive chromatin remodelling activities [8]. DNA methylation modifications are reversible yet heritable, conferring important contributions to epigenetic memory.

Little is known about how DNA methylation is targeted to specific regions. Our group provided evidence that the control of DNA methylation patterns involves the poly(ADP-ribosyl)ation (PARylation) process [9]. PARylation is a post-translational protein modification catalysed by the poly(ADP-ribose) polymerase (PARP) enzymes. By using NAD^+^ as a substrate, PARP enzymes add ADP-ribose polymers (PAR) to a target protein, thus governing its function [9]. PARylation competes with DNA methylation either by directly counteracting the recruitment to DNA and catalytic activity of DNA methyl transferases [10, 11], writer enzymes involved in 5-methyl cytosine (5mC) placement, or by promoting the action of factors that maintain a local chromatin conformation that is refractory to 5mC acquisition [12–14]. In addition, PARylation facilitates active DNA de-methylation by promoting the expression, activity and access to DNA of DNA methylation erasers, such as the Ten-eleven translocation methylcytosine dioxygenase enzymes (TET), which promote *locus*-specific reversal of DNA methylation by catalysing the sequential oxidation of 5mC into 5-hydroxymethylcytosine (5hmC), 5-formylcytosine (5fC) and 5-carboxylcytosine (5caC) [15–19]. In active DNA de-methylation, 5fC and 5caC are replaced by unmodified cytosine via the base-excision repair system, thus resulting in DNA de-methylation [20].

Data from animal models showed that chronic excess of energy intake caused over-activation of PARP enzymes in response to over-production of reactive oxygen species and inflammation [21–27]. PARylation hyperactivation has been associated with dysfunction of glucose metabolism and development of T2DM, while the inhibition or genetic deficiency of PARP enzymes exert protective effects in diabetic rats and mice [28–31].

Notably, studies on a zebrafish model of diabetes have documented that inhibition of PARylation prevents hyperglycaemia-induced DNA de-methylation [32]. However, little is known on the interplay between PARylation processes and DNA methylation alterations in the clinical setting of human diabetes.

Aim of this study was to explore the association between PARylation process and DNA methylation profile in peripheral blood mononuclear cells (PBMC) from T2DM subjects and controls. Firstly, we measured PAR levels and evaluated clinical and biochemical correlates. Then, we investigated global levels of 5mC and of the DNA de-methylation intermediates 5hmC and 5fC in relation to PAR levels and clinical/biochemical parameters. Lastly, we studied whether differential methylation of some candidate genes (*SOCS3*, *SREBF1*, and *TXNIP*), causing dysfunctional effects that impinge on glucose metabolism, was related to PAR levels.

## Results

### Characteristics of the study population

This study involves 61 patients with T2DM and 48 normoglycaemic individuals who make up the control group. The main demographic and clinical features of the study population together to treatment of subjects with oral antidiabetic agents are shown in Table 1.

**Table 1:** Characteristics of the study population

	CT	T2DM	*P*
*N*	48	61	
Age (years)	60 ± 8	63 ± 9	0.065
Male % (*n*)	48 (23)	62 (38)	0.174^1^
Disease duration (years)	–	8.51 ± 7.47	
BMI (kg/m^2^)	26.12 ± 3.77	29.85 ± 5.14	** < 0.001**
Waist circ. (cm)	84 ± 8.1	106.8 ± 11.69	** < 0.001**
DBP (mm Hg)	79.09 ± 7.70	81.33 ± 11.08	0.311
SBP (mm Hg)	124.76 ± 10.39	139.35 ± 18.65	** < 0.001**
FBG (mg/dl)	88.69 ± 10.98	143.13 ± 41.04	** < 0.001**
HbA1c (%)	5.25 ± 0.35	7.58 ± 1.39	** < 0.001**
Total cholesterol (mg/dl)	213.18 ± 35.27	181.86 ± 39.56	** < 0.001**
LDL cholesterol (mg/dl)	124.65 ± 30.82	91.29 ± 45.61	** < 0.001**
HDL cholesterol (mg/dl)	64.41 ± 14.78	46.41 ± 11.41	** < 0.001**
Triglycerides (mg/dl)	100.11 ± 33.53	177.56 ± 83.39	** < 0.001**
ALT (U/l)	19.93 ± 6.4	22.67 ± 13.51	0.184
AST (U/l)	21.37 ± 5.0	19.42 ± 8.49	0.154
GGT (U/l)	16.94 ± 5.79	28.51 ± 21.4	**0.004**
Total bilirubin (mg/dl)	0.57 ± 0.06	0.58 ± 0.27	0.929
Direct bilirubin (mg/dl)	0.19 ± 0.01	0.22 ± 0.008	0.624
Creatinine (mg/dl)	0.89 ± 0.17	0.9 ± 0.32	0.744
MALB (mg/l)	–	24.95 ± 29.61	
PAR (pg/ml)	63.96 ± 38.04	108.38 ± 70.57	**0.001**
5mC (%)	0.566 ± 0.185	0.679 ± 0.246	0.144
5hmC (%)	0.037 ± 0.009	0.070 ± 0.037	** < 0.001**
5fC (%)	0.002 ± 0.002	0.006 ± 0.006	**0.001**
Insulin treated % (*n*)	–	**28 (17)**	
Metformin treated % (*n*)	–	**54 (33)**	
Incretins treated % (*n*)	–	**28 (17)**	

BMI, body mass index; DBP, diastolic blood pressure; SBP, systolic blood pressure; FBG, fasting blood glucose; HbA1c, glycated haemoglobin A1c; ALT, Alanine Transaminase; AST, Aspartate Transaminase; GGT, Gamma-glutamyl Transpeptidase; MALB, microalbuminuria; PAR, poly ADP-ribose; 5mC, 5-methylcytosine; 5hmC, 5-hydroxymethylcytosine; 5fC, 5-formylcytosine

*P* value: Student t-test or Mann–Whitney *U*-test (continuous variables) and chi-square test^1^ (prevalence, for categorical variables). Bold text indicates significant *P* values (≤ 0.05)

Values are mean ± SD for continuous variables; percentage (number) for categorical variables

### Measurement of PAR levels and evaluations of clinical and biochemical correlates

**Fig. 1:**
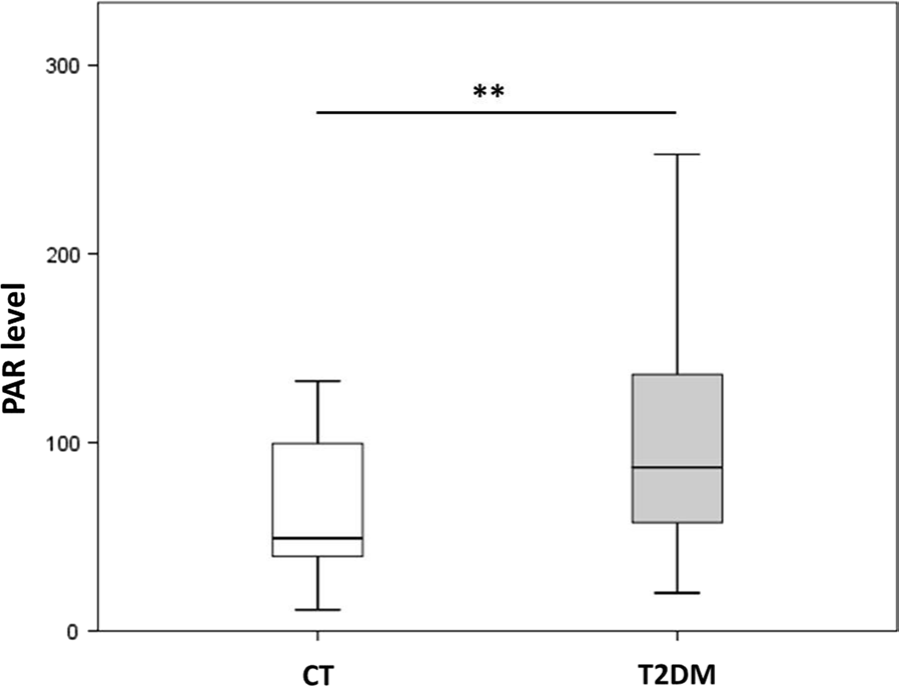
PAR levels in diabetic and control individuals. Box-whisker plot of PAR amount in T2DM patients and control individuals (CT). Boxes show the median, the 25th and the 75th percentiles. Whiskers show the minimum and the maximum data point. **p ≤ 0.01 (Student *t*-test). *N* = 48 CT, 61 T2DM

PAR levels were markedly increased in T2DM patients in comparison with controls (*p* = 0.001) (Table 1 and Fig. 1).

After stratifying the T2DM population in two subgroups of patients with PAR levels above (*high PAR)* or below *(low PAR)* the median PAR value, we found that subjects with high PAR had significantly greater HbA1c levels than those with low PAR (*p* = 0.004) (Additional file 2: Table S1). PAR levels showed a strong positive correlation with HbA1c and a weaker positive correlation with GGT, not confirmed at the Benjamini–Hochberg (BH) false discovery rate (FDR) correction (Additional file 2: Table S2). No association was found between PAR, markers of obesity, presence and/or type of antidiabetic treatment and other metabolic parameters (Additional file 2: Tables S1 and S2).

HbA1C levels remained significantly associated with high PAR levels at the multivariable linear regression analysis after adjustment for age, sex and FBG (*r*^2^ = 0.638, *p* = 0.009, Table 2).

**Table 2:** Multivariate regression analysis. PAR level, considered as a continuous trait, is the dependent variable

	Unst. coeff	Std. coeff	*P*
	B	Beta	
(Constant)	− 155.59		0.032
Age	0.78	0.12	0.371
Gender	7.80	0.05	0.692
FBG	− 0.42	− 0.26	0.117
HbA1c	34.28	0.82	** < 0.001**
GGT	0.42	0.13	0.404
*r*^2^ = 0.638	*P* = 0.009		

*N* = 61 T2DM subjects. Bold text indicates significant *P* values (≤ 0.05)

**Fig. 2:**
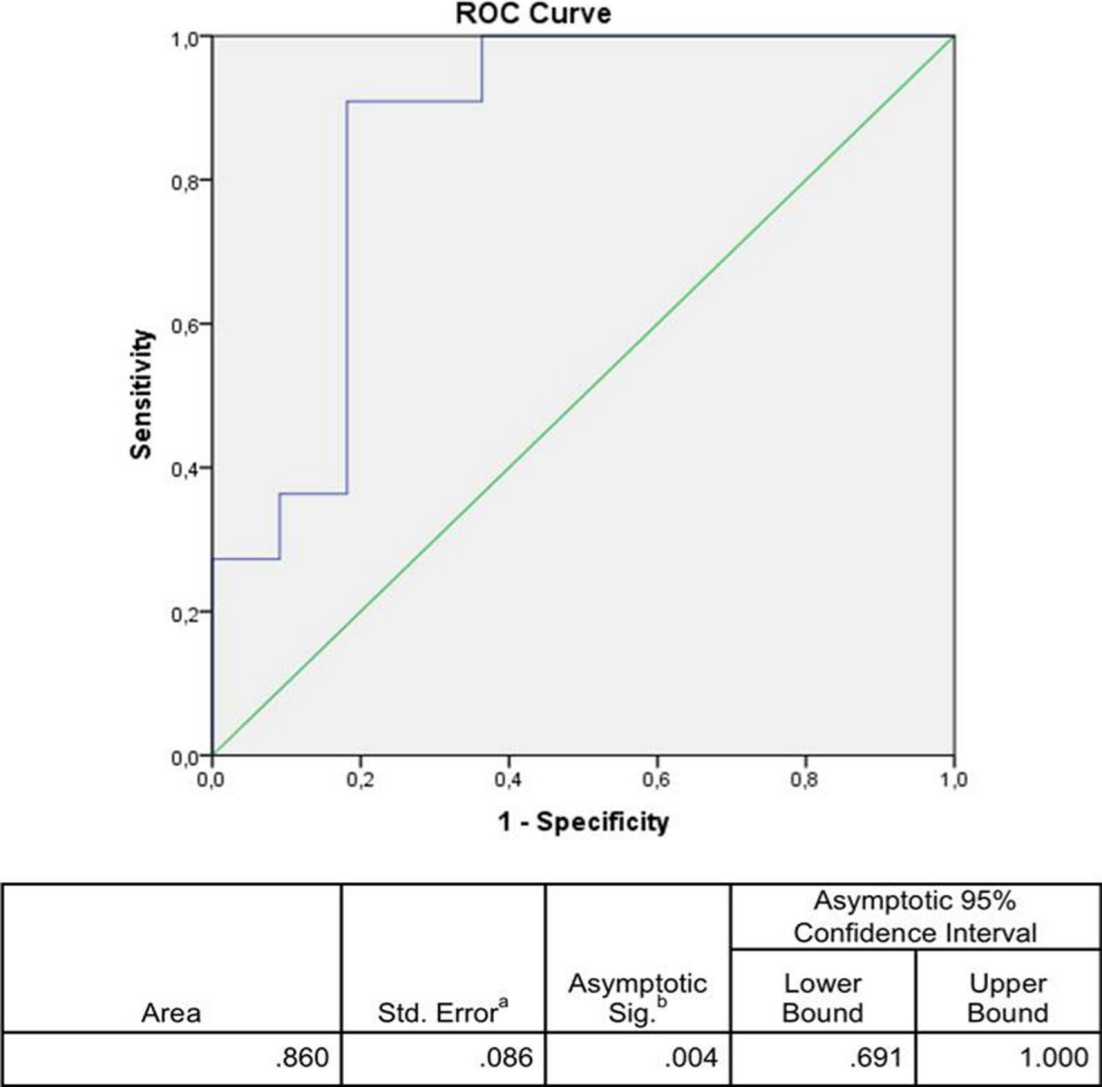
Receiver operating characteristics (ROC) and the corresponding area under the curve (AUC) for HbA1c as predictor for high-PAR levels in T2DM patients. The T2DM group median of PAR level was used as threshold for the binary classification of patients into the low- (*N* = 30) or high-PAR (*N* = 31) categories. ^a^Based on nonparametric assumption. ^b^Null hypothesis: real area = 0.5

Moreover, high PAR levels associated with the presence of increased HbA1c with an AUC = 0.860 (95% CI: 0.691–1.0; *p* = 0.004) at the ROC curve adjusted for age, sex and FBG (Fig. 2).

### Measurement of 5mC, 5hmC and 5fC global levels and evaluation of correlations with clinical and biochemical parameters and with PAR levels

**Fig. 3:**
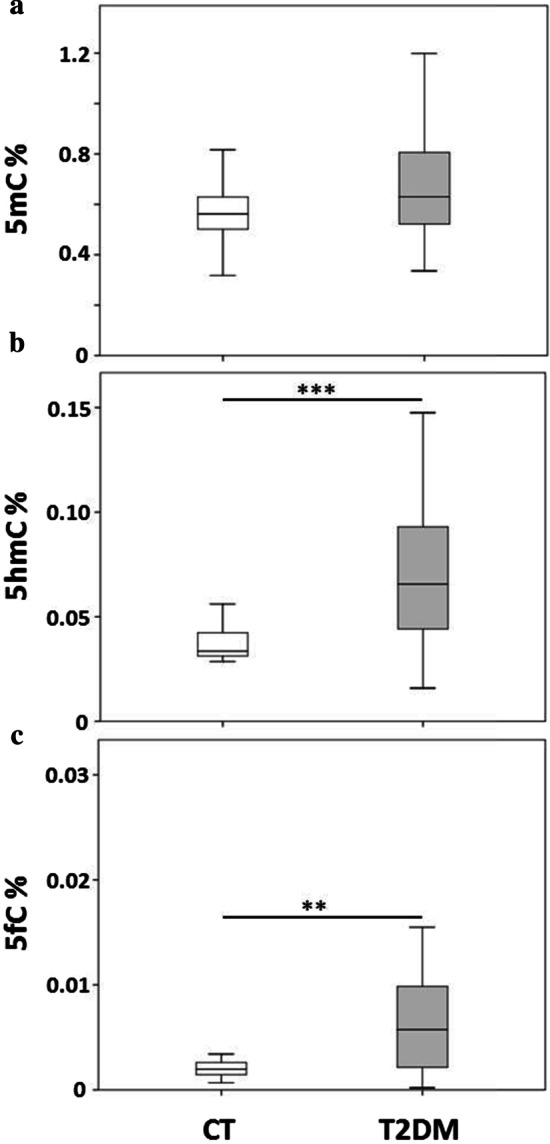
Levels of global 5mC, 5hmC and 5fC in control and T2DM individuals. Box-whisker plots of 5mC (**a**), 5hmC (**b**) and 5fC (**c**) percentage in genomic DNA. Boxes show the median, the 25th and the 75th percentiles. Whiskers show the minimum and the maximum data point. **p ≤ 0.01, ***p ≤ 0.001 (Student t-test). *N* = 48 CT, 61 T2DM

5hmC and 5fC levels were significantly higher in T2DM patients compared to controls (*p *< 0.001, *p* = 0.001, respectively) (Table 1 and Fig. 3).

Stratification analysis showed that T2DM patients with elevated 5hmC had significantly higher HbA1c levels, total and direct bilirubin and PAR levels in comparison to those with low 5hmC levels (Additional file 2: Table S3).

T2DM individuals with high 5fC showed significantly increased FBG and HbA1c levels than those with low 5fC levels (Additional file 2: Table S3).

At the bivariate correlation analysis, 5hmC levels positively correlated with PAR levels, HbA1c, total and direct bilirubin, FBG and triglycerides and negatively correlated with HDL cholesterol (Additional file 2: Table S4). Concerning 5fC, bivariate correlation analyses displayed its positive association with FBG, HbA1c, total bilirubin and PAR levels (Additional file 2: Table S4).

All the above associations shown in Additional file 2: Table S3 and S4 were confirmed by BH correction.

Multivariate regression analysis adjusted for age and sex, identified PAR level and HbA1c as the major determinants of increased 5hmC levels (Table 3, *r*^2^ = 0.89, *p* = 0.002) and 5fC (Table 4, *r*^2^ = 0.58, *p* = 0.009), respectively.

**Table 3:** Multivariate regression analysis. 5hmC level is the dependent variable

	Unst. coeff	Std. coeff	*P*
	B	Beta	
(Constant)	− 2.20E−02		0.738
Age	1.00E−03	0.30	0.131
Gender	− 5.00E−03	− 0.06	0.801
FBG	− 3.01E−04	− 0.32	0.142
HbA1c	5.00E−03	0.25	0.269
HDL	− 1.00E−03	− 0.29	0.286
Triglycerides	1.43E−05	0.03	0.865
Total bilirubin	2.80E−02	0.19	0.343
PAR	3.26E−04	0.61	**0.009**
*r*^2^ = 0.888	*P* = 0.002		

*N* = 61 T2DM subjects. Bold text indicates significant *P* values (≤ 0.05)

**Table 4:** Multivariate regression analysis.5fC level is the dependent variable

	Unst. coeff	Std. coeff	*P*
	B	Beta	
(Constant)	− 7.00E−03		0.205
Age	− 4.64E−05	− 0.112	0.505
Gender	1.00E−03	0.087	0.612
FBG	5.40E−06	0.048	0.803
HbA1c	2.00E−03	0.656	**0.016**
Total bilirubin	3.00E−03	0.185	0.343
PAR	− 1.15E−06	− 0.019	0.938
*r*^2^ = 0.581	*P* = 0.009		

*N* = 61 T2DM subjects. Bold text indicates significant* P* values (≤ 0.05)

### Evaluation of PAR-related differential methylation of candidate genes *SOCS3*, *SREBF1*, and *TXNIP*

**Fig. 4:**
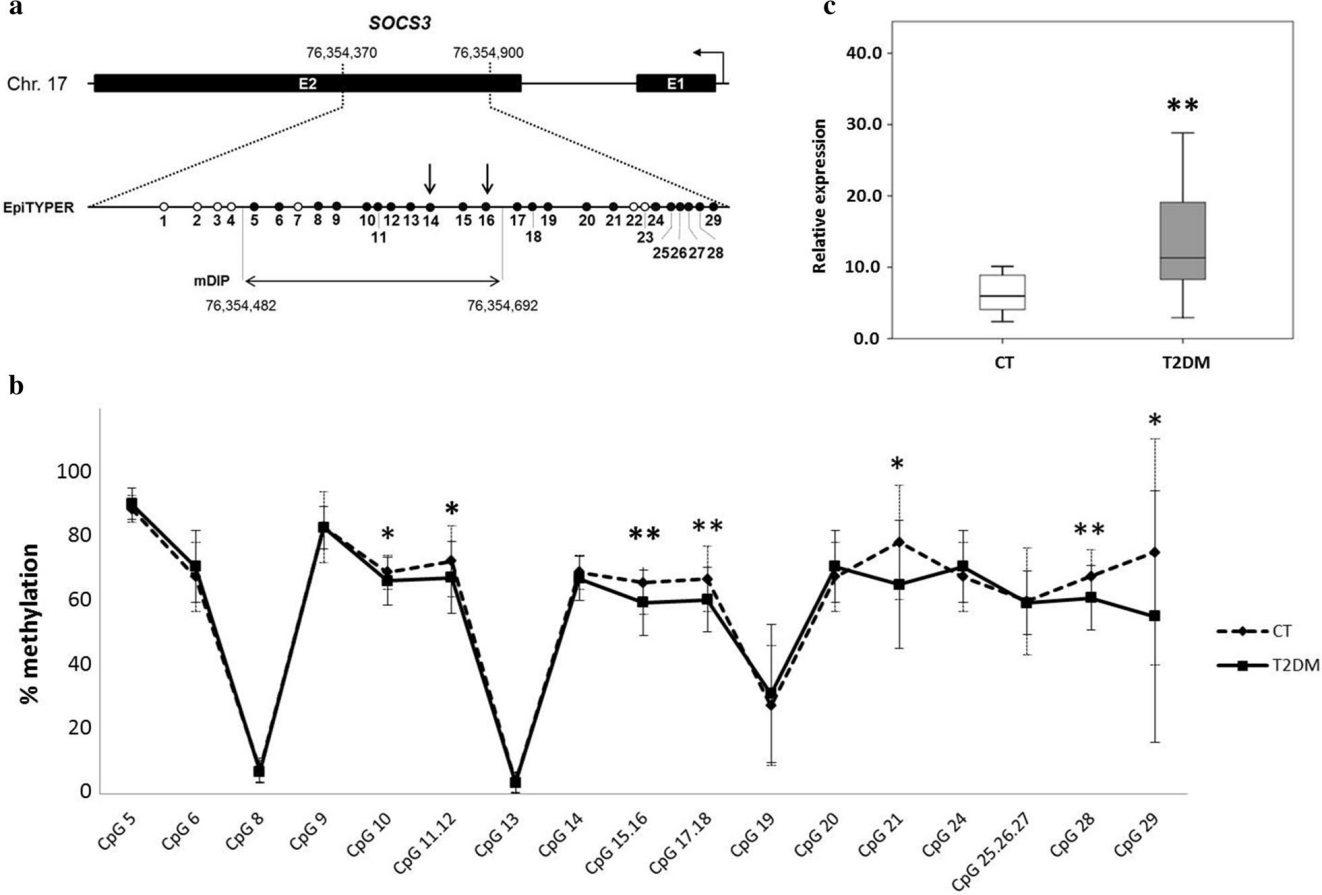
DNA methylation and gene expression profiles of *SOCS3* in control and T2DM individuals. (**a**) Schematic representation of the CpG sites distribution within the DNA region under investigation in the *SOCS3 locus*. The image indicates the location and extent of the region analysed in the EpiTYPER assay (filled and empty circles represent the analysed and unanalysed CpG sites, respectively) and the PCR amplicon used in the modified DNA immunoprecipitation assay (mDIP). The arrows indicate the CpG sites analysed in the methylation-sensitive PCR assay. Filled boxes represent exons. The genomic positions refer to the 2009 (GRCh37/hg19) genome assembly. (**b**) *SOCS3* CpG methylation percentage in T2DM patients and controls. Data are mean ± SD. *p ≤ 0.05, **p ≤ 0.01 (Student t-test). (**c**) Box-whisker plots of *SOCS3* mRNA levels in T2DM patients and controls. Boxes show the median, the 25th and the 75th percentiles. Whiskers show the minimum and the maximum data point. **p ≤ 0.01 (Student t-test). *N* = 48 CT, 61 T2DM

We then evaluated the DNA methylation of three gene *loci* (*SOCS3*, *SREBF1* and *TXNIP*), recently reported to be associated with T2DM and T2DM risk in whole blood epigenome-wide studies [33, 34]. We focused on the microarray probes emerged in these studies and we measured DNA methylation of the target *loci* by mass spectrometry-based bisulfite sequencing (Materials and methods). In our design, cpg18181703 corresponds to CpG 13 in *SOCS3* amplicon (Fig. 4a), cpg11024682 corresponds to CpG 2 in *SREBF1* amplicon (Additional file 1: Figure S1a) and cpg19693031 corresponds to CpG 5 in *TXNIP* amplicon (Additional file 1: Figure S1b). This analysis showed the presence of an altered methylation profile in T2DM patients compared to controls, only for the *SOCS3* gene. Specifically, multiple CpG sites (i.e*.* CpG 10, 11.12, 15.16, 17.18, 21, 28 and 29) showed reduced methylation levels in T2DM patients (Fig. 4b). Parallel gene expression analyses showed a significant upregulation of *SOCS3* transcript levels in T2DM patients compared with controls (Fig. 4c). In contrast, no significant differences were found for the other genes (Additional file 1: Figure S1c–f).

**Fig. 5:**
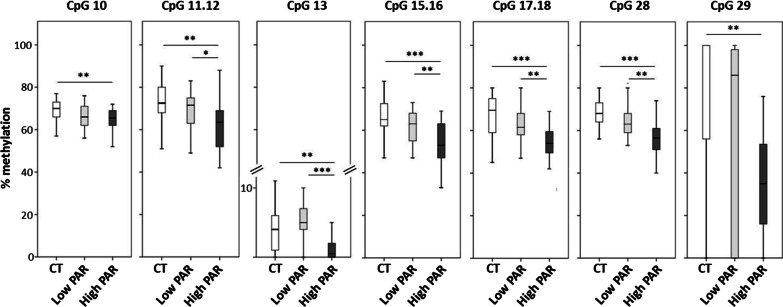
Levels of *SOCS3* CpG methylation in *low* and *high PAR* T2DM patients and in control individuals. Box-whisker plot showing the methylation percentage of different *SOCS3* CpG sites as detected by the EpiTYPER assay. Boxes show the median, the 25th and the 75th percentiles. Whiskers show the minimum and the maximum data point. Comparison between groups was performed by the Kruskal–Wallis test followed by the Dunn-Bonferroni post hoc test for pairwise comparisons. Significant differences are indicated by the asterisks. *p ≤ 0.05, **p ≤ 0.01, ***p ≤ 0.001. N = 48 CT, 30 *low PAR*, 31 *high PAR*

The relationship between *SOCS3* DNA methylation and PAR levels in T2DM patients was then investigated. *High PAR* T2DM patients showed elevated *SOCS3* gene expression and decreased methylation levels—mostly in the CpG sites described above (i.e. CpG 10, 11.12, 15.16, 17.18, 28 and 29) and in one additional site (i.e. CpG 13) (Additional file 2: Table S5). In *high PAR* T2DM subjects, most of these sites showed also a different methylation level compared to *low PAR* T2DM individuals and controls (i.e. CpG 11.12, 13, 15.16, 17.18 and 28) (Additional file 2: Table S5 and Fig. 5). Bivariate correlation analyses carried out in T2DM patients (Additional file 2: Table S6) displayed that increased PAR levels associated with reduced methylation in multiple *SOCS3* CpG sites (*i.e.* CpG 11.12, 13, 15.16, 17.18, 28) and with up-regulated *SOCS3* expression. In addition, the methylation level of most of these sites (*i.e.* CpG 11.12, 13, 28) also correlated negatively with the *SOCS3* gene expression.

The BH correction confirmed most of the associations shown in the Additional file 2: Table S5 and S6, with the exception of the CpG sites 13 and 28 association with SOCS3 gene expression (Additional file 2: Table S6).

### Evaluation of PAR-related differential accumulation of active DNA de-methylation intermediates in the *SOCS3locus*

The association between PAR levels and global accumulation of active DNA de-methylation intermediates 5hmC and 5fC suggested that reduced CpG methylation and upregulation of *SOCS3 * may correlate with increased 5hmC and 5fC in *high PAR* T2DM subjects. Therefore, to confirm this assumption we assessed 5hmC and 5fC amounts in the *SOCS3 locus* of *high PAR and low PAR* T2DM subjects, and controls. We selected 10 subjects for each group, matched by age and gender and determined both the amount of 5hmC *versus* 5mC/unmethylated cytosine for specific CpG sites by MSRD assay and the amount of 5fC *versus* 5hmC within a DNA region encompassing SOCS3 CpG sites 5–16 (Fig. 4a) by using the mDIP assay.

**Fig. 6:**
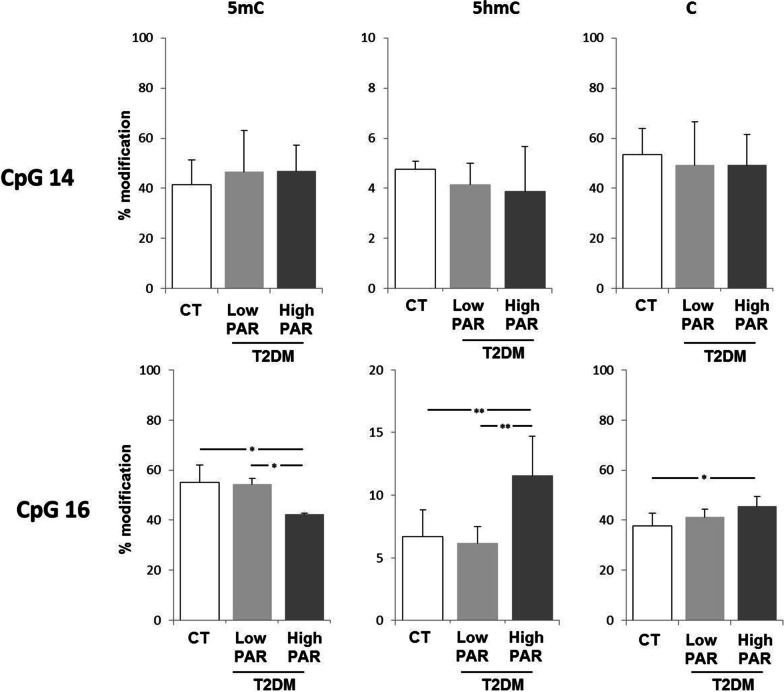
Methylation and hydroxymethylation status of *SOCS3* CpG 14 and 16 in *low* and *high PAR* T2DM patients and in control individuals. Percentage of methylated (5mC), hydroxymethylated (5hmC) and unmethylated (C) cytosine as detected by methylation-sensitive PCR. Data are mean ± SD. Statistically significant differences as detected by ANOVA followed by the Bonferroni post-hoc test are indicated by the asterisks. *p ≤ 0.05, **p ≤ 0.01. N = 10 CT, 10 *low PAR*, 10 *high PAR*

The first analysis involved the CpG 16 (Fig. 4a), part of the CpG unit 15.16 that showed hypomethylation in our cohort of T2DM patients in relation to PAR level and the CpG 14 (Fig. 4a) as an invariant control site showing no methylation changes (Additional file 2: Table S5). As shown in Fig. 6, the results confirmed that CpG 16 was hypomethylated in *high PAR* compared to *low PAR* T2DM patients and controls, showing reduction of 5mC, accumulation of unmethylated cytosine and, moreover, a significant increase in the active de-methylation intermediate 5hmC. As expected, no significant changes in 5mC, 5hmC or unmethylated cytosine were detected in the CpG 14.

**Fig. 7:**
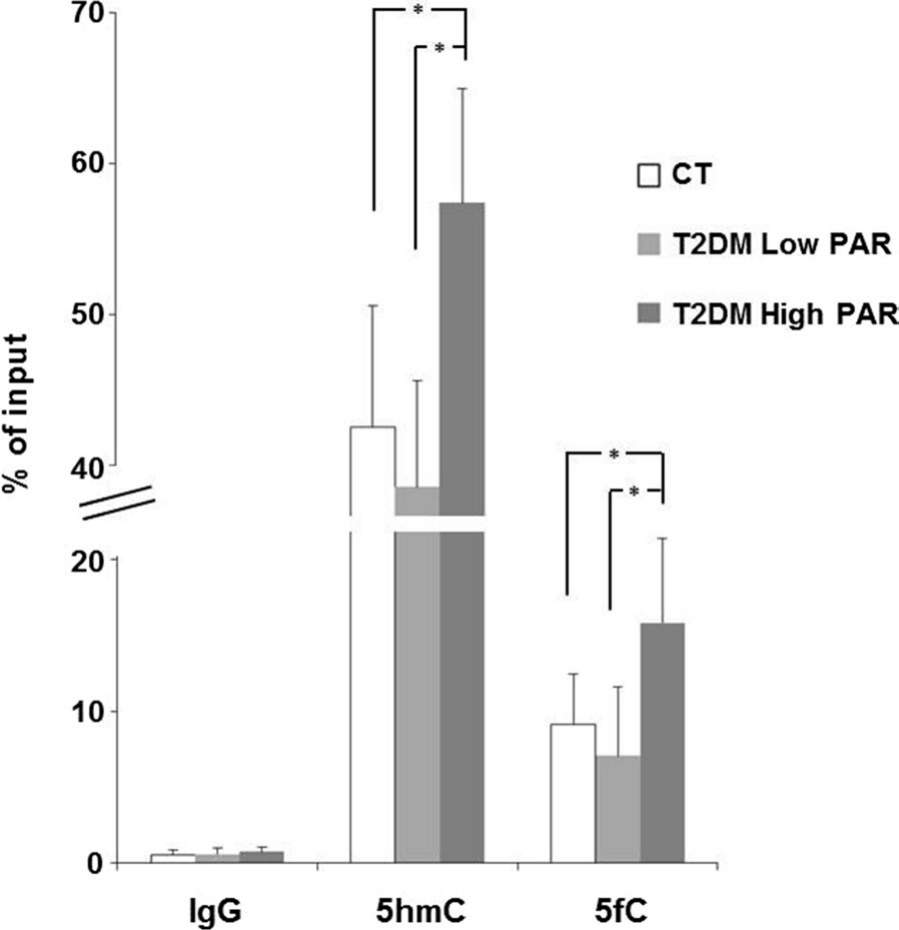
Enrichment of 5hmC and 5fC within *SOCS3 locus* in *low* and *high PAR* T2DM patients and in control individuals. Percent of input detected in 5hmC, 5fC and IgG immunoprecipitated DNA fractions in mDIP experiments. Data are mean ± SD. Statistically significant differences as revealed by ANOVA and the Bonferroni post-hoc tests are indicated by the asterisks. *p ≤ 0.05, **p ≤ 0.01. *N* = 10 CT, 10 *low PAR*, 10 *high PAR*

On the other side, the mDIP analysis evidenced that *high PAR* T2DM patients had significantly higher levels of both 5hmC and 5fC compared to *low PAR* T2DM patients and controls, as shown by a greater immunoprecipitation of 5hmC- and 5fC-rich DNA fragments in this subgroup (Fig. 7).

## Discussion

This study demonstrates that individuals with T2DM have higher PAR levels than age-, sex-comparable metabolically healthy individuals, and that increased PAR is associated with high HbA1c levels independently of potential confounders. Moreover, in our study T2DM patients have greater 5hmC and 5fC levels compared to controls, and these alterations correlate with PAR levels and with poor glyco-metabolic profile in these individuals.

Increased PARylation may interfere with the dynamic balance between methylation and de-methylation, therefore, its upregulation may underpin DNA hypomethylation [35–39] and the increase in hydroxymethylation [40, 41] observed in T2DM individuals with poor glycaemic control. The identification of high PAR levels in our population of T2DM patients is in line with previous data reporting PAR accumulation in blood cells [21] and skin biopsies [22, 24] from T2DM patients, substantiating the view that PARP activity is increased in human diabetes. The association of PAR level with HbA1c matches earlier findings showing a positive correlation between PAR levels in skin biopsies and HbA1c in T2DM patients [24]. This further supports multiple evidence obtained from *in-vitro* and animal studies that identified high glucose as a major factor triggering activation of PARylation in diabetes [25, 30, 32, 42].

In contrast to previous findings [24], we did not detect an association between PAR and fasting glucose levels. This discrepancy may depend on the tissue-dependent dynamics of PARylation processes in response to fluctuations in blood glucose[43].

In this study, we found higher global levels of the de-methylation intermediates 5hmC and 5fC in T2DM patients. This result is in line with previous evidence obtained in both blood [40, 44] and cardiac cells [41] from subjects with T2DM. Given that 5hmC and 5fC serve as consecutive intermediates in the 5mC turnover process, their accumulation suggests that an active DNA de-methylation process may occur in presence of T2DM. Moreover, 5hmC and 5fC share a positive association with HbA1c and FBG, pointing towards the existence of a close association between poor glycaemic control and changes in the epigenome in patients with diabetes. Of note, high 5hmC levels are also associated with low HDL cholesterol and high triglycerides, suggesting a potential specific relationship between this de-methylation intermediate and the presence of an altered lipid profile.

The detection of PAR-related accumulation of 5hmC and 5fC agrees with our previous observations describing PARylation as a negative regulator of DNA methylation. From this point of view, the increase of these 5mC oxidative intermediates could be indicative of an ongoing process of active DNA de-methylation induced by upregulated PARylation, as previously reported in physiological [15, 19, 45] and pathological [32] contexts.

Alterations of global DNA methylation profile may be at the basis of key dynamic epigenetic states that impair the expression of diabetes-related genes, finally resulting in the manifestation of pro-inflammatory, insulin-resistant phenotypes. In this context, we evaluated the differential methylation pattern of the candidate genes *SOCS3*, *SREBF1*, and *TXNIP* between T2DM patients and controls, and found the presence of an altered methylation profile only for the *SOCS3* gene. However, we cannot rule out that part of the EpiTyper signal for *SREBF1* and *TXNIP* genes may hide changes in 5hmC levels. In fact, this assay is based on treatment of DNA with bisulphite, which cannot discriminate between 5mC and 5hmC. In addition, non-findings for *SREBF1* and *TXNIP* genes with respect to previous reports may be also ascribed to location effects and differences in cohort size and composition.

Focusing on *SOCS3* gene, increased PAR levels associated with reduced methylation in multiple *SOCS3* CpG sites and with up-regulated *SOCS3* expression levels. Lastly, our results demonstrated that *high PAR* T2DM subjects had increased 5hmC and 5fC in *SOCS3* (CpG 16 site) in comparison with *low PAR* T2DM subjects and controls.

Predominant hypomethylation of *SOCS3* in T2DM subjects was inversely associated with both PAR and *SOCS3* transcript levels, suggesting that increased PARylation may mediate between hypomethylation and upregulation of *SOCS3* expression. On the other hand, the accumulation of 5hmC and 5fC supports the possibility that hypomethylation of *SOCS3* is due to an active de-methylation process in *high PAR* T2DM subjects.

The finding that *SOCS3* methylation levels in *low* PAR T2DM patients did not differ from those measured in healthy controls suggests that such epigenetic changes are related to PARylation dynamics -which may occur in T2DM mostly in presence of poor glycaemic control- and not to diabetes per se.

There are several clinical implications deriving from the finding of altered *SOCS3* methylation in T2DM individuals. First, *SOCS3* is an important negative regulator of the insulin signalling pathway and *SOCS3* upregulation has been involved in insulin resistance [46–50]. Moreover, hypomethylation of *SOCS3* associated with T2DM risk and mediated the effect of important environmental factors such as obesity, sedentary time and stress [33, 34, 51–54].

This study provides some new evidence on epigenetic processes occurring in diabetes. Investigating causes of alterations of DNA methylation in T2DM can provide relevant information for understanding the basis of disease and for the identification of novel therapeutic approaches and prevention strategies.

Our work has some limitations. First, this is an exploratory study with a cross-sectional design, and a cause-and-effect relationship in our study findings cannot be established with certainty. Further studies are required to elucidate the molecular aspects linking unbalanced PARylation to DNA de-methylation in T2DM. Furthermore, analyses for this study were carried out in PBMC, as altered PARylation [21, 23, 55] and changes of methylation pattern [33, 34, 56, 57] have been both demonstrated in these cells in presence of T2DM. However, we acknowledge that the use of PBMC may provide partial information on the overall DNA methylation profile, which is known to vary in relation to the cell type. Nevertheless, mounting evidence indicates that diabetes-related epigenetic changes in immune blood cells are associated with chronic inflammation [58] and partly mirror the deteriorated metabolic function of diabetes-relevant tissues [59].

## Conclusions

This work demonstrates the existence of a relationship between DNA methylation alterations in T2DM and hyper-activation of PARylation, via activation of the DNA de-methylation pathway, and links these molecular alterations to impaired glycaemic control.

Although new research paths in the pursuit of mechanisms underlying epigenetic deregulation in T2DM are needed, this finding may have significant implications on clinical practice. In the foreseeable future, specific epigenetically defined subgroups of T2DM patients could be identified through PAR detection and benefit from the use of PARP inhibitors as “epigenetic drugs” for disease treatment.

## Methods

### Study population

This case–control study involved 109 male and female participants in the age range of 31–86 years comprising 61 patients with T2DM and 48 age- and sex-matched healthy individuals as controls. The enrolment of diabetic subjects was carried out at the Diabetes Outpatient Clinic of the Umberto I Hospital, Sapienza University of Rome, Italy. The enrolment of controls was carried out at the Blood Donor Unit of the same Hospital.

### Clinical evaluation

Study participants underwent medical history collection, clinical work-up and fasting blood sampling for routine biochemistry and experimental evaluations. Weight and height were measured with light clothes and without shoes and the body mass index (BMI, kg/m^2^) was then calculated. Waist circumference (cm) was measured midway between the 12th rib and the iliac crest. Systemic systolic and diastolic blood pressure (SBP, DBP, mmHg) were measured after 5-min resting; three measurements were taken and the average of the second and third measurements was recorded and entered in the analyses. Diabetes mellitus has been diagnosed according to the American Diabetes Association 2020 criteria [60]. 53 out of 61 T2DM patients were under treatment with oral glucose controlling agents, such as metformin (54%), incretins (28%), and/or with insulin (28%).

### Laboratory evaluations

Venous blood samples were collected after 12-h fasting for measuring blood glucose (FBG, mg/dl), glycosylated haemoglobin (HbA1c, mmol/mol, %), total cholesterol (mg/dl), high-density lipoprotein cholesterol (HDL, mg/dl), triglycerides (mg/dl), aspartate aminotransferase (AST, IU/l), alanine aminotransferase (ALT, IU/l), gamma-glutamyl transpeptidase (GGT, mg/dl), total bilirubin (mg/dl), conjugated bilirubin (mg/dl) and creatinine (mg/dl) by centralized standard methods. Low-density lipoprotein (LDL, mg/dl) cholesterol value was obtained using the Friedewald formula.

Morning urine samples were collected for albuminuria assessment (mg/dl).

### Isolation of PBMC

Venous blood samples (6 ml) were collected in vacutainer EDTA- containing tubes. PBMC were isolated by density gradient centrifugation using the Lymphoprep™ − 1.077 g/mL solution (Axis-Shield) according to the instructions of the manufacturer. The isolated cells were immediately processed or stored at − 80°C as pellets.

### Assessment of PAR concentration in PBMC

In order to determine the net concentration of PAR, nuclear extracts were prepared from fresh PBMC samples and processed for PAR quantification by the use of the HT PARP in vitro Pharmacodynamic Assay II (Trevigen) following the instructions of the manufacturer. Collection of the chemiluminescent signal was performed by using the Victor X light plate reader (Perkin Elmer Inc.). PAR concentrations were calculated by using the standard curve method and a standard sample for PAR concentration supplied by the kit. The standard curve ranged from 10 t 1000 pg/ml. The *r*^2^ value for the linear curve fit was 0.993. In each experimental plate, samples were measured in duplicate and Jurkat cell lysates were used as an inter-run calibration sample.

### Isolation of total DNA and quantification of global 5mC, 5hmC and 5fC content

Total DNA was isolated from PBMC by using the DNeasy Blood and Tissue Kit (Qiagen) following the instructions of the manufacturer. Purified DNA was used to detect global content of 5mC (MethylFlash methylated DNA quantification kit, Epigentek), 5hmC (MethylFlash hydroxymethylated DNA quantification kit, Epigentek) and 5fC (MethylFlash 5-Formylcytosine (5-fC) DNA Quantification Kit). Collection of the chemiluminescent signal was performed by using the Victor X light plate reader (Perkin Elmer Inc.). The content of each cytosine modification in the samples was determined by using the standard curve method and a standard DNA sample supplied by the kit. In each experimental plate, samples were measured in duplicate and positive DNA control samples, supplied by the manufacturer, were used as inter-run calibration samples.

### DNA methylation profiling of SREBF1, SOCS3 and TXNIP gene loci by the EpiTYPER assay

The methylation profiling of selected DNA regions in the *SREBF1, SOCS3 and TXNIP* genes was determined by the EpiTYPER assay (Sequenom), which measures the methylation ratio of the specific CpG sites or groups of adjacent CpG sites, termed CpG units. Genomic coordinates (GRCh37/hg19 assembly) of the analysed DNA regions are given in Fig. 4a and Additional file 1: Figure S1A,B. PBMC DNA (500 ng) was bisulfite-converted using the EZ-96 DNA Methylation Kit (Zymo Research) with the following modifications: the incubation in the CT buffer was performed for 21 cycles of 15 min at 55 °C and 30 s at 95 °C; the elution of bisulfite-treated DNA was performed with 100 μl of water. PCR amplifications were performed using the following bisulfite-specific pairs, designed by the EpiDesigner online software: *SREBF1* FP aggaagagagAGGAGGTATAGATTTTGGGTTATGG, RP cagtaatacgactcactatagggagaaggctATAAAAAACTCCCTCTTCCAAAAAA; *SOCS3* FP aggaagagagGTTTGTTATATTTTGTAGGGAGAGGG, RP cagtaatacgactcactatagggagaaggctACCCAATCTAAAACCAAAAACCTAC; *TXNIP* FP aggaagagagAATAGTTTTTGTAATGGAGTGTGGG, RP cagtaatacgactcactatagggagaaggctAAAACAATTACTACTACTTTAAAAACCAAA.

Amplicons were then processed according to EpiTYPER standard protocol. In each experimental plate, samples were measured in duplicate.

### CpG-specific quantification of 5hmC in the SOCS3 locus by methyl-sensitive restriction digestion (MSRD)

The CpG-specific analysis of 5hmC content in the *SOCS3 locus* was performed by the EpiMark 5hmC and 5mC analysis kit (New England Biolabs), which is based on Glucosyl-5-hmC (5-ghmC)-sensitive restriction endonucleases. The assay discriminates between 5-ghmC and 5mC in the CpG site present in the target sequence (CCGG) of the methylation-sensitive restriction enzymes MspI and HpaII. The level of DNA methylation is then inferred from its differential susceptibility to restriction by MspI and HpaII after glucosylation of 5-hmC. MspI can cut DNA when cytosine, 5mC or 5hmC is present at the recognition site. Conversely, the digestion is blocked when 5hmC is modified by the glucose addition. By contrast, HpaII activity is blocked by all these modifications, so it only cuts in the presence of unmethylated cytosine. The *SOCS3* CpG sites available to this analysis are shown in Fig. 4a (i.e. the CpGs 14 and 16).

The analysis was performed according to the manufacturer protocol. Briefly, purified total PBMC DNA was subjected to 5hmC glucosylation reaction for 16 h at 37 °C. Subsequently, the sample underwent digestion with either the HpaII or the MspI for 16 h at 37 °C. After that, digested samples were diluted in water and used as templates for quantitative PCR reactions performed by using the SYBR green MasterMix (BioLabs) and primer pairs recognizing the *SOCS3 locus* DNA regions containing the HpaII/MspI restriction sites of interest. The sequence of primer pairs used was as follows: CpG 14 FP CGAGAAGATCCCCCTGGTGT, RP TGACGGTCTTCCGACAGAGA;

CpG 16 FP CGTCTGCCCAGCCACTC, RP ACACCAGGGGGATCTTCTCG. In each experimental PCR plate, samples were measured in triplicate.

### Quantification of 5fC and 5hmC in the SOCS3 locus by DNA immuno-precipitation (mDIP)

Purified PBMC DNA was subjected to sonication (40% amplitude; 0.5 cycle, UP100H ultrasonic processor, Hielscher) to obtain fragments about 500–300 bp. Subsequently, samples were heat-denatured for 10 min at 95 °C and cooled on ice for 10 min. After dilution in IP buffer (10 mM Na-Phosphate buffer pH 7.0, 0.14 M NaCl, 0.05% Triton X-100), aliquots of the samples (2.5 µg) were used for immuno-precipitation by the addition of 1 μg of anti-5hmC, anti-5fC (Active Motif) antibodies or normal rabbit/mouse IgG as control. The samples were then incubated for 16 h at 4 °C on a rotating platform. Immunocomplexes were recovered by the addition of 45 μl of the Protein-A or G agarose beads (Millipore), incubation for 2 h on a rotating platform at 4 °C and washing in the IP buffer. DNA was isolated from the samples by standard proteinase K digestion /phenol–chloroform, extraction/ethanol precipitation. The obtained DNA was then subjected to qPCR amplification by using the SYBR green MasterMix (BioLabs) and primer pairs recognizing the *SOCS3 locus* (Fig. 4a). The primer pair sequence was the following: FP: TATTACATCTACTCCGGGGGC, RP: GCAGCTGGGTGACTTTCTCAT. In each experimental PCR plate samples were measured in triplicate.

### Gene expression analysis by reverse transcription–quantitative PCR (RT-qPCR)

Frozen PBMC pellets were thawed on ice and processed for RNA extraction and DNAse I digestion by using the RNeasy Mini Kit (Qiagen) according to the instructions of the manufacturer. RNA concentration, purity and integrity were evaluated as previously described [61]. Reverse transcription was carried out using the SuperScript VILO cDNA Synthesis Kit (Life Technologies) on equal amounts of total RNA (1 μg).

*SREBF1*, *SOCS3* and *TXNIP* mRNA levels were determined by quantitative PCR performed by using the SYBR green MasterMix (BioLabs). Gene expression was quantified according to the relative calibrator normalized quantification method using the *Hypoxanthine Phosphoribosyltransferase 1* (*HPRT1*) gene transcript as reference for normalization. An inter-run calibration sample was used in each plate to correct for technical variance between runs and to compare results from different plates. The calibrator consisted of cDNA prepared from HEK293T cells. In each experimental PCR plate, samples were measured in triplicate. The primers used in the assay were as follows: *SREBF1* FP ACCAGCGTCTACCATAGCCCT, RP CATTGAGCAGCCAGACCACT; *SOCS3* FP GGAGACTTCGATTCGGGACC, RP GGAGCCAGCGTGGATCTG; *TXNIP* FP TGTTCATTCCTGATGGGCGG, RP GCTTTGGGGACCACAATTCG; *HPRT-1* FP TTGGAAAGGGTGTTTATTCCTCA, RP TCCAGCAGGTCAGCAAAGAA.

### Statistical analysis

All measurements were performed at least in triplicate for every participant and average values were used for the analyses. For continuous variables, normal distribution of data was verified by the Kolmogorov–Smirnov and Shapiro–Wilk normality tests.

Values are given as mean ± standard deviation (SD), median (interquartile range, IQ) or percentage, as appropriate. Groups were formed by aggregating individuals based on the presence of T2DM and bundling together the T2DM patients in two consecutive categories based on having levels of PAR, 5hmC or 5fC above or below the median value. Comparisons between two independent groups were performed by the Mann–Whitney test (when not normally distributed) or the Student t-test (when normally distributed) for continuous variables and *χ*^2^ test for categorical parameters. Comparisons between more than two subgroups were performed by the Kruskal–Wallis test, followed by pairwise comparisons by the Dunn-Bonferroni method. Bivariate correlations were explored by Pearson or Spearman (when not normally distributed variables are involved) r coefficients.

In order to control for false discovery rate (FDR), the Benjamini–Hochberg procedure was applied at a FDR = 0.25.

Multivariable linear regression models were built to investigate the relationship of PAR, 5hmC or 5fC levels (entered as continuous dependent variables) with sex, age and metabolic parameters (entered as independent variables). The predictive value of PAR levels on the presence of T2DM was explored by receiver operating characteristic (ROC) curve adjusted for sex, age and potential confounders. All statistical analyses were carried out using SPSS software (IBM SPSS Statistics Version 23.0).

## Supplementary Information

**Additional file 1.** Supplementary Figure (Figure S1).

**Additional file 2.** Supplentary Tables (Tables S1–S6).

## Data Availability

Data and materials are available on request from the authors.

## Abbreviations

T2DM: Type 2 diabetes mellitus
SBP: Systolic blood pressure
DBP: Diastolic blood pressure
BMI: Body Mass Index

## References

[1] Cho NH, Shaw JE, Karuranga S, Huang Y, da Rocha Fernandes JD, Ohlrogge AW (2018). IDF Diabetes Atlas: global estimates of diabetes prevalence for 2017 and projections for 2045. Diabetes Res Clin Pract..

[2] Pirola L, Balcerczyk A, Okabe J, El-Osta A (2010). Epigenetic phenomena linked to diabetic complications. Nat Rev Endocrinol..

[3] Suzuki MM, Bird A (2008). DNA methylation landscapes: provocative insights from epigenomics. Nat Rev Genet..

[4] Davegårdh C, García-Calzón S, Bacos K, Ling C (2018). DNA methylation in the pathogenesis of type 2 diabetes in humans. Mol Metab..

[5] Ling C, Rönn T (2019). Epigenetics in human obesity and type 2 diabetes. Cell Metab..

[6] Rosen ED, Kaestner KH, Natarajan R, Patti M-E, Sallari R, Sander M (2018). Epigenetics and epigenomics: implications for diabetes and obesity. Diabetes.

[7] Wahl S, Drong A, Lehne B, Loh M, Scott WR, Kunze S (2017). Epigenome-wide association study of body mass index, and the adverse outcomes of adiposity. Nature.

[8] Du Q, Luu P-L, Stirzaker C, Clark SJ (2015). Methyl-CpG-binding domain proteins: readers of the epigenome. Epigenomics.

[9] Ciccarone F, Zampieri M, Caiafa P (2017). PARP1 orchestrates epigenetic events setting up chromatin domains. Semin Cell Dev Biol..

[10] Reale A, De Matteis G, Galleazzi G, Zampieri M, Caiafa P (2005). Modulation of DNMT1 activity by ADP-ribose polymers. Oncogene.

[11] Zampieri M, Passananti C, Calabrese R, Perilli M, Corbi N, De Cave F (2009). Parp1 localizes within the Dnmt1 promoter and protects its unmethylated state by its enzymatic activity. PLoS ONE.

[12] Guastafierro T, Catizone A, Calabrese R, Zampieri M, Martella O, Bacalini MG (2013). ADP-ribose polymer depletion leads to nuclear Ctcf re-localization and chromatin rearrangement. Biochem J..

[13] Guastafierro T, Cecchinelli B, Zampieri M, Reale A, Riggio G, Sthandier O (2008). CCCTC-binding factor activates PARP-1 affecting DNA methylation machinery. J Biol Chem..

[14] Zampieri M, Guastafierro T, Calabrese R, Ciccarone F, Bacalini MG, Reale A (2012). ADP-ribose polymers localized on Ctcf-Parp1-Dnmt1 complex prevent methylation of Ctcf target sites. Biochem J..

[15] Ciccarone F, Klinger FG, Catizone A, Calabrese R, Zampieri M, Bacalini MG (2012). Poly(ADP-ribosyl)ation acts in the DNA demethylation of mouse primordial germ cells also with DNA damage-independent roles. PLoS ONE.

[16] Ciccarone F, Valentini E, Bacalini MG, Zampieri M, Calabrese R, Guastafierro T (2014). Poly(ADP-ribosyl)ation is involved in the epigenetic control of TET1 gene transcription. Oncotarget.

[17] Ciccarone F, Valentini E, Zampieri M, Caiafa P (2015). 5mC-hydroxylase activity is influenced by the PARylation of TET1 enzyme. Oncotarget.

[18] Fujiki K, Shinoda A, Kano F, Sato R, Shirahige K, Murata M (2013). PPARγ-induced PARylation promotes local DNA demethylation by production of 5-hydroxymethylcytosine. Nat Commun..

[19] Hajkova P, Jeffries SJ, Lee C, Miller N, Jackson SP, Surani MA (2010). Genome-Wide Reprogramming in the Mouse Germ Line Entails the Base Excision Repair Pathway. Science (80-)..

[20] Wu X, Zhang Y (2017). TET-mediated active DNA demethylation: mechanism, function and beyond. Nat Rev Genet..

[21] Adaikalakoteswari A, Rema M, Mohan V, Balasubramanyam M (2007). Oxidative DNA damage and augmentation of poly(ADP-ribose) polymerase/nuclear factor-kappa B signaling in patients with type 2 diabetes and microangiopathy. Int J Biochem Cell Biol..

[22] Altaf QA, Ali A, Piya MK, Raymond NT, Tahrani AA (2016). The relationship between obstructive sleep apnea and intra-epidermal nerve fiber density, PARP activation and foot ulceration in patients with type 2 diabetes. J Diabetes Complic..

[23] Giorgi A, Tempera I, Napoletani G, Drovandi D, Potestà C, Martire S (2017). Poly(ADP-ribosylated) proteins in mononuclear cells from patients with type 2 diabetes identified by proteomic studies. Acta Diabetol..

[24] Szabo C, Zanchi A, Komjati K, Pacher P, Krolewski AS, Quist WC (2002). Poly(ADP-Ribose) polymerase is activated in subjects at risk of developing type 2 diabetes and is associated with impaired vascular reactivity. Circulation.

[25] Du X, Matsumura T, Edelstein D, Rossetti L, Zsengeller Z, Szabo C (2003). Inhibition of GAPDH activity by poly(ADP-ribose) polymerase activates three major pathways of hyperglycemic damage in endothelial cells. J Clin Invest..

[26] Obrosova IG, Drel VR, Pacher P, Ilnytska O, Wang ZQ, Stevens MJ (2005). Oxidative-nitrosative stress and poly(ADP-ribose) polymerase (PARP) activation in experimental diabetic neuropathy: the relation is revisited. Diabetes.

[27] Szabo C (2009). Role of nitrosative stress in the pathogenesis of diabetic vascular dysfunction. Br J Pharmacol..

[28] Burkart V, Wang ZQ, Radons J, Heller B, Herceg Z, Stingl L (1999). Mice lacking the poly(ADP-ribose) polymerase gene are resistant to pancreatic beta-cell destruction and diabetes development induced by streptozocin. Nat Med..

[29] Szabo C (2005). Roles of poly(ADP-ribose) polymerase activation in the pathogenesis of diabetes mellitus and its complications. Pharmacol Res..

[30] Szabo C, Biser A, Benko R, Bottinger E, Susztak K (2006). Poly(ADP-ribose) polymerase inhibitors ameliorate nephropathy of type 2 diabetic Leprdb/db mice. Diabetes.

[31] Virag L, Szabo C (2002). The therapeutic potential of poly(ADP-ribose) polymerase inhibitors. Pharmacol Rev..

[32] Dhliwayo N, Sarras MP, Luczkowski E, Mason SM, Intine RV (2014). Parp inhibition prevents ten-eleven translocase enzyme activation and hyperglycemia-induced DNA demethylation. Diabetes.

[33] Dayeh T, Tuomi T, Almgren P, Perfilyev A, Jansson PA, de Mello VD (2016). DNA methylation of loci within ABCG1 and PHOSPHO1 in blood DNA is associated with future type 2 diabetes risk. Epigenetics.

[34] Chambers JC, Loh M, Lehne B, Drong A, Kriebel J, Motta V (2015). Epigenome-wide association of DNA methylation markers in peripheral blood from Indian Asians and Europeans with incident type 2 diabetes: a nested case-control study. Lancet Diabetes Endocrinol..

[35] Luttmer R, Spijkerman AM, Kok RM, Jakobs C, Blom HJ, Serne EH (2013). Metabolic syndrome components are associated with DNA hypomethylation. Obes Res Clin Pract..

[36] Martín-Núñez GM, Rubio-Martín E, Cabrera-Mulero R, Rojo-Martínez G, Olveira G, Valdés S (2014). Type 2 diabetes mellitus in relation to global LINE-1 DNA methylation in peripheral blood: a cohort study. Epigenetics.

[37] Thongsroy J, Patchsung M, Mutirangura A (2017). The association between Alu hypomethylation and severity of type 2 diabetes mellitus. Clin Epigenet..

[38] Volkmar M, Dedeurwaerder S, Cunha DA, Ndlovu MN, Defrance M, Deplus R (2012). DNA methylation profiling identifies epigenetic dysregulation in pancreatic islets from type 2 diabetic patients. EMBO J..

[39] Pirola L, Balcerczyk A, Tothill RW, Haviv I, Kaspi A, Lunke S (2011). Genome-wide analysis distinguishes hyperglycemia regulated epigenetic signatures of primary vascular cells. Genome Res..

[40] Pinzon-Cortes JA, Perna-Chaux A, Rojas-Villamizar NS, Diaz-Basabe A, Polania-Villanueva DC, Jacome MF (2017). Effect of diabetes status and hyperglycemia on global DNA methylation and hydroxymethylation. Endocr Connect..

[41] Spallotta F, Cencioni C, Atlante S, Garella D, Cocco M, Mori M (2018). Stable oxidative cytosine modifications accumulate in cardiac mesenchymal cells from type2 diabetes patients: rescue by alpha-ketoglutarate and TET-TDG functional reactivation. Circ Res..

[42] Garcia Soriano F, Virag L, Jagtap P, Szabo E, Mabley JG, Liaudet L (2001). Diabetic endothelial dysfunction: the role of poly(ADP-ribose) polymerase activation. Nat Med..

[43] Horvath EM, Benko R, Kiss L, Muranyi M, Pek T, Fekete K (2009). Rapid “glycaemic swings” induce nitrosative stress, activate poly(ADP-ribose) polymerase and impair endothelial function in a rat model of diabetes mellitus. Diabetologia.

[44] Yuan EF, Yang Y, Cheng L, Deng X, Chen SM, Zhou X (2019). Hyperglycemia affects global 5-methylcytosine and 5-hydroxymethylcytosine in blood genomic DNA through upregulation of SIRT6 and TETs. Clin Epigenetics..

[45] Surani MA, Hajkova P (2010). Epigenetic reprogramming of mouse germ cells toward totipotency. Cold Spring Harb Symp Quant Biol..

[46] Emanuelli B, Peraldi P, Filloux C, Sawka-Verhelle D, Hilton D, Van Obberghen E (2000). SOCS-3 is an insulin-induced negative regulator of insulin signaling. J Biol Chem..

[47] Jorgensen SB, O’Neill HM, Sylow L, Honeyman J, Hewitt KA, Palanivel R (2013). Deletion of skeletal muscle SOCS3 prevents insulin resistance in obesity. Diabetes.

[48] Sachithanandan N, Fam BC, Fynch S, Dzamko N, Watt MJ, Wormald S (2010). Liver-specific suppressor of cytokine signaling-3 deletion in mice enhances hepatic insulin sensitivity and lipogenesis resulting in fatty liver and obesity. Hepatology.

[49] Shi H, Cave B, Inouye K, Bjorbaek C, Flier JS (2006). Overexpression of suppressor of cytokine signaling 3 in adipose tissue causes local but not systemic insulin resistance. Diabetes.

[50] Torisu T, Sato N, Yoshiga D, Kobayashi T, Yoshioka T, Mori H (2007). The dual function of hepatic SOCS3 in insulin resistance in vivo. Genes Cells..

[51] Al Muftah WA, Al-Shafai M, Zaghlool SB, Visconti A, Tsai PC, Kumar P (2016). Epigenetic associations of type 2 diabetes and BMI in an Arab population. Clin Epigenetics..

[52] Liu X, Qian X, Tu R, Mao Z, Huo W, Zhang H (2020). SOCS3 methylation mediated the effect of sedentary time on type 2 diabetes mellitus: The Henan Rural Cohort study. Nutr Metab Cardiovasc Dis..

[53] Mathur R, Hui Q, Huang Y, Gwinn M, So-Armah K, Freiberg MS (2019). DNA methylation markers of type 2 diabetes mellitus among male veterans with or without human immunodeficiency virus infection. J Infect Dis..

[54] Xu K, Zhang X, Wang Z, Hu Y, Sinha R (2018). Epigenome-wide association analysis revealed that SOCS3 methylation influences the effect of cumulative stress on obesity. Biol Psychol..

[55] Tempera I, Cipriani R, Campagna G, Mancini P, Gatti A, Guidobaldi L (2005). Poly(ADP-ribose)polymerase activity is reduced in circulating mononuclear cells from type 2 diabetic patients. J Cell Physiol..

[56] Florath I, Butterbach K, Heiss J, Bewerunge-Hudler M, Zhang Y, Schottker B (2016). Type 2 diabetes and leucocyte DNA methylation: an epigenome-wide association study in over 1,500 older adults. Diabetologia.

[57] Toperoff G, Aran D, Kark JD, Rosenberg M, Dubnikov T, Nissan B (2012). Genome-wide survey reveals predisposing diabetes type 2-related DNA methylation variations in human peripheral blood. Hum Mol Genet..

[58] Naidoo V, Naidoo M, Ghai M (2018). Cell- and tissue-specific epigenetic changes associated with chronic inflammation in insulin resistance and type 2 diabetes mellitus. Scand J Immunol..

[59] Gillberg L, Ling C (2015). The potential use of DNA methylation biomarkers to identify risk and progression of type 2 diabetes. Front Endocrinol..

[60] American Diabetes Association. 2. Classification and diagnosis of diabetes: standards of medical care in diabetes-2020. Diabetes Care. 2020 Jan;43(Suppl 1):S14–31. 10.2337/dc20-S002 31862745

[61] Verdone L, La Fortezza M, Ciccarone F, Caiafa P, Zampieri M, Caserta M (2015). Poly(ADP-ribosyl)ation affects histone acetylation and transcription. PLoS ONE.

