# Supplementary material for: Increased PARylation impacts the DNA methylation process in type 2 diabetes mellitus
Source: Clin Epigenetics. 2021 May 17;13:114. doi: 10.1186/s13148-021-01099-1 (PMC8130175; doi:10.1186/s13148-021-01099-1)

*Figure S1*. DNA methylation and gene expression profiles of *SREBF1* and *TXNIP* in control and T2DM individuals. (a, b) Schematic representation of the CpG sites distribution within the DNA regions under investigation in the *SREBF1* (a) and *TXNIP* (b) *loci*. The images indicate the location and extent of the regions analysed in the EpiTYPER assay (filled circles represent the analysed CpG sites). Filled boxes represent exons. The genomic positions refer to the 2009 (GRCh37/hg19) assembly. c, d) *SREBF1* (c) and *TXNIP* (d) CpG methylation percentage in T2DM patients and controls. Data are mean ± SD. **p* ≤ 0.05, ***p* ≤ 0.01 (Student t-test). e, f) Box-whisker plots of *SREBF1* (e) and *TXNIP* (f) mRNA levels in T2DM patients and controls. Boxes show the median, the 25th and the 75^th^ percentiles. Whiskers show the minimum and the maximum data point. N= 48 CT, 61 T2DM.


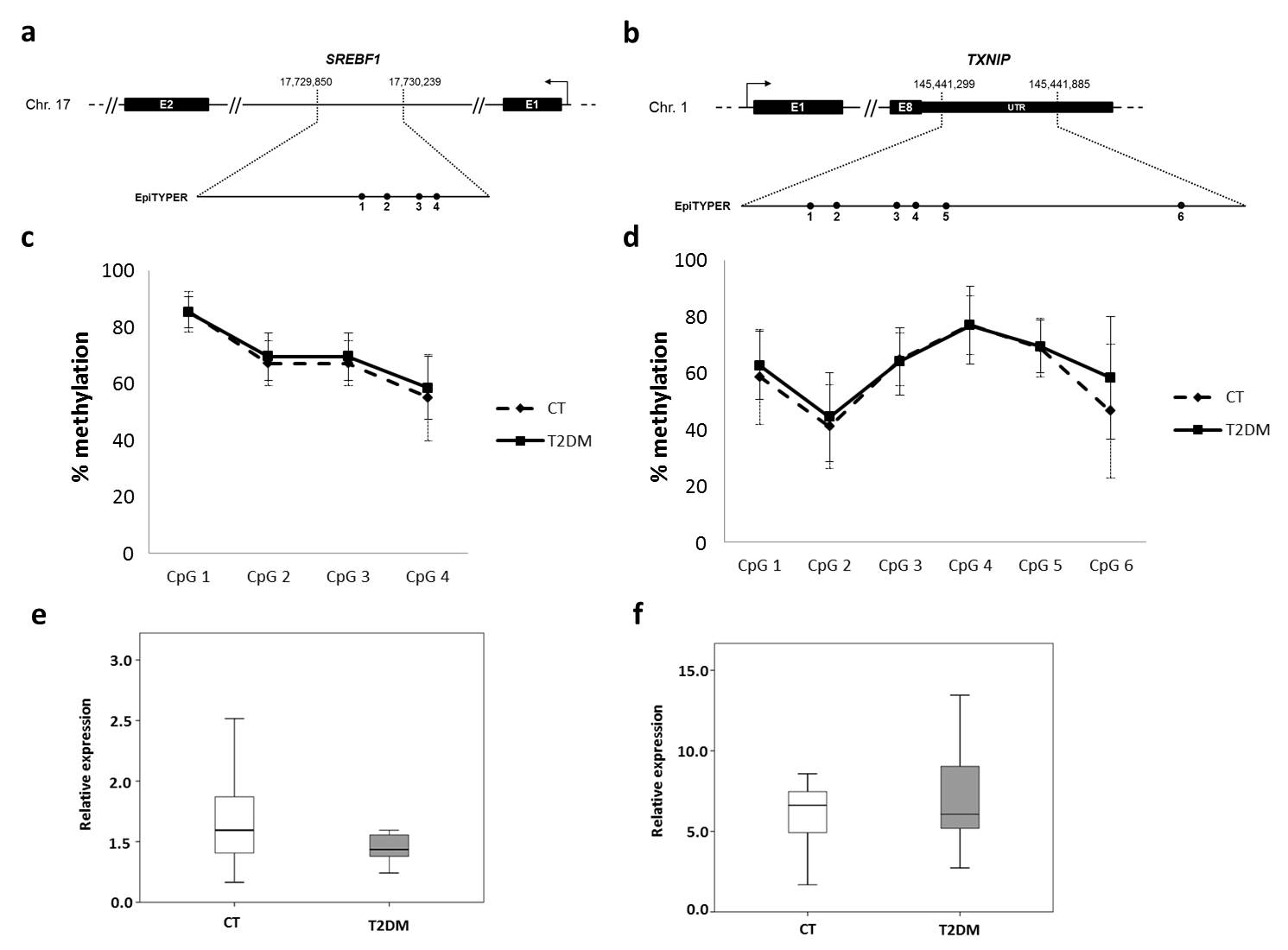

Supplement: Supplementary file 1 — Additional file 1. Supplementary Figure (Figure S1). [file 13148_2021_1099_MOESM1_ESM.docx]
