# Supplementary material for: Increased PARylation impacts the DNA methylation process in type 2 diabetes mellitus
Source: Clin Epigenetics. 2021 May 17;13:114. doi: 10.1186/s13148-021-01099-1 (PMC8130175; doi:10.1186/s13148-021-01099-1)
Supplement: Supplementary file 2 — Additional file 2. Supplentary Tables (Tables S1–S6). [file 13148_2021_1099_MOESM2_ESM.docx]

*Table S1.* Clinical and biochemical characteristics of the study population in relation to PAR levels.

|  | **Low PAR (N=30)** | | |  | **High PAR (N=31)** | | | ***P*** |  |
| --- | --- | --- | --- | --- | --- | --- | --- | --- | --- |
| **PAR (pg/ml)** | 55.07 | ± | 17.72 |  | 161.69 | ± | 62.72 | **-** |  |
| **Age (years)** | 62.32 | ± | 12.76 |  | 64.92 | ± | 11.49 | 0.453 |  |
| **Male %** | 67 | | |  | 57 | | | 0.556 | ^1^ |
| **Disease duration (years)** | 7.17 | ± | 4.39 |  | 8.65 | ± | 9.65 | 0.621 |  |
| **BMI (kg/m^2^)** | 30.36 | ± | 5.17 |  | 29.74 | ± | 5.23 | 0.704 |  |
| **Waist circ. (cm)** | 107.61 | ± | 11.58 |  | 105.80 | ± | 12.76 | 0.774 |  |
| **DBP (mm Hg)** | 81.84 | ± | 9.89 |  | 83.33 | ± | 11.11 | 0.658 |  |
| **SBP (mm Hg)** | 138.68 | ± | 16.90 |  | 144.90 | ± | 20.73 | 0.308 |  |
| **FBG (mg/dl)** | 138.08 | ± | 43.16 |  | 145.96 | ± | 40.68 | 0.514 |  |
| **HbA1c (%)** | 7.02 | ± | 0.91 |  | 8.21 | ± | 1.71 | **0.004*** |  |
| **Total cholesterol (mg/dl)** | 177.21 | ± | 35.07 |  | 186.05 | ± | 43.92 | 0.489 |  |
| **LDL cholesterol (mg/dl)** | 88.87 | ± | 44.43 |  | 92.18 | ± | 52.26 | 0.821 |  |
| **HDL cholesterol (mg/dl)** | 46.00 | ± | 10.53 |  | 46.33 | ± | 11.48 | 0.925 |  |
| **Triglycerides (mg/dl)** | 176.79 | ± | 87.56 |  | 171.52 | ± | 84.76 | 0.848 |  |
| **ALT (U/l)** | 24.38 | ± | 12.81 |  | 25.04 | ± | 14.86 | 0.869 |  |
| **AST (U/l)** | 19.09 | ± | 5.62 |  | 21.75 | ± | 10.7 | 0.294 |  |
| **GGT (U/l)** | 27.38 | ± | 16.50 |  | 33.86 | ± | 28.11 | 0.441 |  |
| **Total bilirubin (mg/dl)** | 0.61 | ± | 0.27 |  | 0.57 | ± | 0.28 | 0.634 |  |
| **Direct bilirubin (mg/dl)** | 0.22 | ± | 0.07 |  | 0.21 | ± | 0.08 | 0.668 |  |
| **Creatinine (mg/dl)** | 0.93 | ± | 0.23 |  | 0.81 | ± | 0.26 | 0.123 |  |
| **MALB (mg/l)** | 22.39 | ± | 28.97 |  | 22.54 | ± | 29.71 | 0.987 |  |
| **Metformin treated %** | 60.00 | | |  | 48.39 | | | 0.444 | ^1^ |
| **Insulin treated %** | 23.33 | | |  | 32.22 | | | 0.570 | ^1^ |
| **Incretins treated %** | 26.66 | | |  | 29.03 | | | 1.000 | ^1^ |

Stratification was formed by aggregating the T2DM patients in two consecutive categories (low vs high) based on having PAR levels above or below the median value.

Values are mean ± SD for continuous variables; percentage for categorical variables^1^. Pairwise comparisons were performed by Student t - test (continuous variables) and chi-square test^1^ (prevalence, for categorical variables).

Bold text indicates significant P values (≤ 0.05). The asterisk (*) indicates significant difference after the Benjamini-Hochberg correction procedure at a false discovery rate (FDR) of 0.25.

*Table S2***.** Bivariate correlation analysis between PAR levels and clinical and biochemical characteristics in T2DM patients.

|  | **r coeff.** | ***P*** |
| --- | --- | --- |
| **Age (years)** | 0.058 | 0.688 |
| **Gender** | 0.054 | 0.712 |
| **Disease duration (years)** | -0.086 | 0.642 |
| **BMI (kg/m^2^)** | 0.034 | 0.833 |
| **Waist circ. (cm)** | 0.088 | 0.719 |
| **DBP (mm Hg)** | 0.183 | 0.259 |
| **SBP (mm Hg)** | 0.080 | 0.625 |
| **FBG (mg/dl)** | 0.179 | 0.218 |
| **HbA1c (%)** | 0.667 | **<0.001*** |
| **Total cholesterol (mg/dl)** | 0.285 | 0.074 |
| **LDL cholesterol (mg/dl)** | 0.164 | 0.280 |
| **HDL cholesterol (mg/dl)** | -0.077 | 0.637 |
| **Triglycerides (mg/dl)** | 0.015 | 0.928 |
| **ALT (U/l)** | 0.163 | 0.274 |
| **AST (U/l)** | 0.192 | 0.195 |
| **GGT (U/l)** | 0.361 | **0.050** |
| **Total bilirubin (mg/dl)** | 0.141 | 0.367 |
| **Direct bilirubin (mg/dl)** | 0.118 | 0.451 |
| **Creatinine (mg/dl)** | -0.194 | 0.196 |
| **MALB (mg/l)** | -0.097 | 0.539 |
| **Metformin** | 0.136 | 0.317 |
| **Insulin** | 0.067 | 0.621 |
| **Incretins** | -0.086 | 0.523 |

r = Pearson coefficient. N = 61 T2DM subjects. Bold text indicates significant P values (≤ 0.05). The asterisk (*) indicates significant correlation after the Benjamini-Hochberg correction procedure at a FDR = 0.25.

*Table S3*. Clinical and biochemical characteristics of the study population in relation to 5hmC and 5fC levels.

|  | **5hmC** | | | | | | | |  | **5fC** | | | | | | | |  |
| --- | --- | --- | --- | --- | --- | --- | --- | --- | --- | --- | --- | --- | --- | --- | --- | --- | --- | --- |
|  | **Low (N=30)** | | |  | **High (N=31)** | | | ***P*** |  | **Low (N=30)** | | |  | **High (N=31)** | | | ***P*** |  |
| **Age (years)** | 63.15 | ± | 10.50 |  | 67.50 | ± | 11.25 | 0.310 |  | 66.13 | ± | 9.52 |  | 66.63 | ± | 12.47 | 0.903 |  |
| **Male %** | 46.15 | | |  | 51.53 | | | 0.182 | ^2^ | 53.33 | | |  | 68.75 | | | 0.379 | ^1^ |
| **Disease duration (years)** | 7.56 | ± | 5.03 |  | 7.09 | ± | 6.20 | 0.858 |  | 10.36 | ± | 6.34 |  | 6.17 | ± | 4.43 | 0.780 |  |
| **BMI (kg/m^2^)** | 29.99 | ± | 4.54 |  | 30.29 | ± | 5.50 | 0.899 |  | 30.56 | ± | 5.87 |  | 28.42 | ± | 4.33 | 0.305 |  |
| **Waist circ. (cm)** | 104.75 | ± | 6.34 |  | 109.50 | ± | 19.09 | 0.785 |  | 107.67 | ± | 3.06 |  | 105.00 | ± | 15.59 | 0.786 |  |
| **DBP (mm Hg)** | 76.25 | ± | 7.91 |  | 80.91 | ± | 12.00 | 0.354 |  | 81.00 | ± | 9.07 |  | 77.31 | ± | 10.73 | 0.392 |  |
| **SBP (mm Hg)** | 139.38 | ± | 11.48 |  | 134.82 | ± | 11.42 | 0.403 |  | 141.50 | ± | 9.44 |  | 135.23 | ± | 11.65 | 0.180 |  |
| **FBG (mg/dl)** | 127.92 | ± | 34.08 |  | 150.07 | ± | 40.62 | 0.149 |  | 122.87 | ± | 34.65 |  | 154.00 | ± | 38.86 | **0.028*** |  |
| **HbA1c (%)** | 6.85 | ± | 1.52 |  | 8.73 | ± | 1.53 | **0.004*** |  | 6.83 | ± | 0.99 |  | 8.54 | ± | 1.80 | **0.003*** |  |
| **Total cholesterol (mg/dl)** | 182.91 | ± | 40.84 |  | 180.10 | ± | 49.51 | 0.888 |  | 193.17 | ± | 42.33 |  | 168.82 | ± | 43.08 | 0.186 |  |
| **LDL cholesterol (mg/dl)** | 92.18 | ± | 43.21 |  | 81.89 | ± | 53.00 | 0.602 |  | 83.88 | ± | 52.85 |  | 76.63 | ± | 48.50 | 0.704 |  |
| **HDL cholesterol (mg/dl)** | 49.82 | ± | 11.44 |  | 41.70 | ± | 11.51 | 0.122 |  | 50.25 | ± | 13.49 |  | 44.45 | ± | 11.07 | 0.275 |  |
| **Triglycerides (mg/dl)** | 182.91 | ± | 93.88 |  | 197.36 | ± | 83.58 | 0.707 |  | 201.18 | ± | 84.93 |  | 180.31 | ± | 86.83 | 0.559 |  |
| **ALT (U/l)** | 18.36 | ± | 7.38 |  | 28.43 | ± | 17.49 | 0.067 |  | 21.60 | ± | 13.49 |  | 24.54 | ± | 15.37 | 0.595 |  |
| **AST (U/l)** | 17.55 | ± | 4.93 |  | 24.29 | ± | 12.89 | 0.090 |  | 20.64 | ± | 10.72 |  | 21.14 | ± | 9.77 | 0.898 |  |
| **GGT (U/l)** | 21.80 | ± | 12.02 |  | 45.00 | ± | 34.26 | 0.129 |  | 30.44 | ± | 22.94 |  | 32.38 | ± | 30.08 | 0.883 |  |
| **Total bilirubin (mg/dl)** | 0.43 | ± | 0.19 |  | 0.70 | ± | 0.24 | **0.006*** |  | 0.43 | ± | 0.18 |  | 0.65 | ± | 0.30 | **0.029*** |  |
| **Direct bilirubin (mg/dl)** | 0.18 | ± | 0.08 |  | 0.25 | ± | 0.07 | **0.035*** |  | 0.18 | ± | 0.07 |  | 0.24 | ± | 0.08 | **0.041*** |  |
| **Creatinine (mg/dl)** | 0.78 | ± | 0.25 |  | 0.83 | ± | 0.15 | 0.547 |  | 0.82 | ± | 0.21 |  | 0.84 | ± | 0.26 | 0.802 |  |
| **MALB (mg/l)** | 22.02 | ± | 35.98 |  | 14.68 | ± | 15.92 | 0.528 |  | 12.84 | ± | 23.02 |  | 23.91 | ± | 32.30 | 0.318 |  |
| **PAR (pg/ml)** | 86.44 | ± | 35.38 |  | 199.20 | ± | 60.86 | **< 0.001*** |  | 106.41 | ± | 59.64 |  | 159.09 | ± | 81.39 | 0.062 |  |
| **5hmC %** | 0.040 | ± | 0.016 |  | 0.099 | ± | 0.027 | ***-*** | ^1^ | 0.053 | ± | 0.030 |  | 0.087 | ± | 0.037 | **0.016*** |  |
| **5fC %** | 0.004 | ± | 0.004 |  | 0.010 | ± | 0.006 | **0.010*** | ^1^ | 0.002 | ± | 0.001 |  | 0.011 | ± | 0.005 | - |  |
| **Metformin treated %** | 66.67 | | |  | 41.93 | | | 0.073 |  | 66.67 | | |  | 41.93 | | | 0.073 | ^1^ |
| **Insulin treated %** | 23.33 | | |  | 32.25 | | | 0.570 |  | 26.66 | | |  | 29.03 | | | 1.000 | ^1^ |
| **Incretins treated %** | 30.00 | | |  | 25.80 | | | 0.780 |  | 23.33 | | |  | 32.25 | | | 0.570 | ^1^ |

Strata were formed by aggregating the T2DM patients in two consecutive categories (low vs high) based on having a level of 5hmC or 5fC above or below the T2DM group median of the variables.

Values are mean ± SD for continuous variables; percentage (number) for categorical variables^1^.

P-value: Student t test (continuous variables) and chi-square test^1^ (prevalence, for categorical variables). Bold text indicates significant P values (≤ 0.05). The asterisk (*) indicates significant difference after the Benjamini-Hochberg correction procedure at a FDR = 0.25.

*Table S4*. Bivariate correlation analysis between 5hmC and 5fC levels and clinical and biochemical characteristics in T2DM patients.

|  | **5hmC** | |  | **5fC** | |
| --- | --- | --- | --- | --- | --- |
|  | **r coeff.** | ***P*** |  | **r coeff.** | ***P*** |
| **5hmC %** | **-** | ***-*** |  | 0.54 | **< 0.001*** |
| **5fC %** | 0.54 | **0.004*** |  | **-** | ***-*** |
| **PAR (pg/ml)** | 0.71 | **< 0.001*** |  | 0.49 | **0.002*** |
| **Age (years)** | 0.30 | 0.055* |  | 0.17 | 0.279 |
| **Gender** | -0.24 | 0.131 |  | -0.05 | 0.736 |
| **Disease duration (years)** | 0.24 | 0.176 |  | 0.11 | 0.507 |
| **BMI (kg/m^2^)** | -0.01 | 0.779 |  | -0.29 | 0.137 |
| **Waist circ. (cm)** | 0.17 | 0.593 |  | 0.10 | 0.753 |
| **DBP (mm Hg)** | 0.06 | 0.772 |  | -0.05 | 0.813 |
| **SBP (mm Hg)** | -0.09 | 0.667 |  | -0.14 | 0.459 |
| **FBG (mg/dl)** | 0.50 | **0.002*** |  | 0.50 | **0.001*** |
| **HbA1c (%)** | 0.61 | **< 0.001*** |  | 0.61 | **< 0.001*** |
| **Total cholesterol (mg/dl)** | -0.13 | 0.496 |  | -0.11 | 0.536 |
| **LDL cholesterol (mg/dl)** | -0.14 | 0.433 |  | -0.13 | 0.434 |
| **HDL cholesterol (mg/dl)** | -0.45 | **0.013*** |  | -0.11 | 0.558 |
| **Triglycerides (mg/dl)** | 0.41 | **0.024*** |  | 0.05 | 0.786 |
| **ALT (U/l)** | 0.14 | 0.433 |  | 0.07 | 0.692 |
| **AST (U/l)** | 0.01 | 0.939 |  | -0.03 | 0.878 |
| **GGT (U/l)** | 0.25 | 0.279 |  | 0.00 | 0.997 |
| **Total bilirubin (mg/dl)** | 0.58 | **0.001*** |  | 0.36 | **0.047*** |
| **Direct bilirubin (mg/dl)** | 0.40 | **0.042*** |  | 0.30 | 0.110 |
| **Creatinine (mg/dl)** | 0.13 | 0.460 |  | -0.04 | 0.812 |
| **MALB (mg/l)** | 0.09 | 0.673 |  | 0.12 | 0.537 |
| **Metformin** | 0.15 | 0.413 |  | 0.27 | 0.101 |
| **Insulin** | -0.08 | 0.673 |  | 0.44 | 0.795 |
| **Incretins** | 0.19 | 0.294 |  | 0.27 | 0.105 |

r = Spearman’s coefficient. N = 61 T2DM subjects. Bold text indicates significant P values (≤ 0.05). The asterisk (*) indicates significant correlation after the Benjamini-Hochberg correction procedure at a FDR = 0.25.

*Table S5*. Variation of *SOCS3* CpG methylation in diabetic patients in relation to PAR levels (*low* *PAR*, *high PAR* T2DM) and controls.

|  | **Control (N=48)** | | | | |  | **T2DM (N=61)** | | | |  |  | **T2DM low PAR (N=30)** | | | | | | **T2DM high PAR (N=31)** | | | | |  |  |
| --- | --- | --- | --- | --- | --- | --- | --- | --- | --- | --- | --- | --- | --- | --- | --- | --- | --- | --- | --- | --- | --- | --- | --- | --- | --- |
| **Stat** | ***a*** | | | |  |  | ***-*** | | | |  |  |  | ***b*** | | | |  | ***c*** | | | |  |  |  |
|  | **Median** |  |  |  |  |  | **Median** |  | **IQ** |  |  |  |  | **Median** |  | **IQ** |  |  | **Median** |  | **IQ** |  |  |  |  |
|  | **Mean** |  | **SD** |  |  |  | **Mean** |  | **SD** |  | ***P* KW** | ***P* GLM** |  | **Mean** |  | **SD** |  |  | **Mean** |  | **SD** |  |  | ***P* KW** | ***P* GLM** |
| **Expression** | 5.96 | ( | 4.83 | ) | **^b,c^** |  | 11.31 | ( | 12.73 | ) | **0.001*** | **0.023*** |  | 7.75 | ( | 6.76 | ) | **^a,c^** | 13.86 | ( | 11.98 | ) | **^a,b^** | **0.001*** | **< 0.001*** |
|  | 6.72 | ± | 3.67 |  |  |  | 15.03 | ± | 11.46 |  |  |  |  | 8.61 | ± | 4.12 |  |  | 15.17 | ± | 6.41 |  |  |  |  |
| **DNA methylation** |  |  |  |  |  |  |  |  |  |  |  |  |  |  |  |  |  |  |  |  |  |  |  |  |  |
| **CpG 5** | 89.00 | ( | 7.00 | ) |  |  | 91.00 | ( | 6.00 | ) | 0.153 | 0.175 |  | 89.50 | ( | 6.00 | ) |  | 92.00 | ( | 10.00 | ) |  | 0.312 | 0.328 |
|  | 88.67 | ± | 4.16 |  |  |  | 90.35 | ± | 4.98 |  |  |  |  | 89.41 | ± | 4.81 |  |  | 90.94 | ± | 5.69 |  |  |  |  |
| **CpG 6** | 70.50 | ( | 20.00 | ) |  |  | 69.00 | ( | 11.00 | ) | 0.991 | 0.918 |  | 69.00 | ( | 11.00 | ) |  | 68.00 | ( | 17.00 | ) |  | 0.604 | 0.137* |
|  | 67.67 | ± | 10.78 |  |  |  | 70.86 | ± | 11.13 |  |  |  |  | 67.60 | ± | 8.05 |  |  | 74.70 | ± | 14.57 |  |  |  |  |
| **CpG 8** | 8.00 | ( | 4.00 | ) |  |  | 7.00 | ( | 5.00 | ) | 0.391 | 0.292 |  | 7.00 | ( | 4.00 | ) |  | 5.50 | ( | 6.00 | ) |  | 0.417 | 0.260 |
|  | 7.64 | ± | 3.69 |  |  |  | 6.96 | ± | 3.57 |  |  |  |  | 7.41 | ± | 4.06 |  |  | 6.11 | ± | 3.14 |  |  |  |  |
| **CpG 9** | 86.00 | ( | 12.00 | ) |  |  | 84.00 | ( | 7.00 | ) | 0.451 | 0.388 |  | 83.50 | ( | 10.00 | ) |  | 84.00 | ( | 5.00 | ) |  | 0.358 | 0.266 |
|  | 83.11 | ± | 10.99 |  |  |  | 83.02 | ± | 6.57 |  |  |  |  | 81.59 | ± | 7.55 |  |  | 84.50 | ± | 6.25 |  |  |  |  |
| **CpG 10** | 70.00 | ( | 7.00 | ) | **^c^** |  | 67.00 | ( | 8.00 | ) | **0.044*** | **0.05*** |  | 66.00 | ( | 9.00 | ) |  | 65.50 | ( | 7.00 | ) | ^a^ | **0.029*** | **0.031*** |
|  | 69.04 | ± | 5.39 |  |  |  | 66.33 | ± | 7.53 |  |  |  |  | 66.73 | ± | 7.64 |  |  | 63.78 | ± | 7.30 |  |  |  |  |
| **CpG 11.12** | 72.50 | ( | 12.00 | ) | **^c^** |  | 70.00 | ( | 15.00 | ) | 0.056* | **0.01*** |  | 71.50 | ( | 12.00 | ) | **^c^** | 63.50 | ( | 17.00 | ) | ^a, b^ | **0.012*** | **0.003*** |
|  | 72.50 | ± | 11.06 |  |  |  | 67.43 | ± | 11.19 |  |  |  |  | 69.09 | ± | 10.20 |  |  | 62.61 | ± | 11.77 |  |  |  |  |
| **CpG 13** | 4.00 | ( | 5.00 | ) | **^c^** |  | 3.00 | ( | 6.00 | ) | 0.732 | 0.519 |  | 5.00 | ( | 3.00 | ) | **^c^** | 0.50 | ( | 2.00 | ) | ^a, b^ | **< 0.001*** | **< 0.001*** |
|  | 3.79 | ± | 3.14 |  |  |  | 3.54 | ± | 3.24 |  |  |  |  | 5.29 | ± | 2.51 |  |  | 1.22 | ± | 1.56 |  |  |  |  |
| **CpG 14** | 70.00 | ( | 7.00 | ) |  |  | 67.00 | ( | 8.00 | ) | 0.110 | 0.193 |  | 66.50 | ( | 9.00 | ) |  | 66.00 | ( | 7.00 | ) |  | 0.105 | 0.178 |
|  | 69.04 | ± | 5.39 |  |  |  | 67.14 | ± | 6.90 |  |  |  |  | 67.18 | ± | 7.88 |  |  | 65.44 | ± | 5.48 |  |  |  |  |
| **CpG 15.16** | 65.00 | ( | 10.50 | ) | **^c^** |  | 61.00 | ( | 15.00 | ) | **0.011*** | **0.001*** |  | 63.00 | ( | 13.00 | ) | **^c^** | 53.00 | ( | 16.00 | ) | ^a, b^ | **0.001*** | **< 0.001*** |
|  | 65.79 | ± | 9.68 |  |  |  | 59.65 | ± | 10.22 |  |  |  |  | 61.68 | ± | 7.82 |  |  | 53.50 | ± | 10.89 |  |  |  |  |
| **CpG 17.18** | 69.50 | ( | 16.00 | ) | **^c^** |  | 61.00 | ( | 13.00 | ) | **0.009*** | **0.008*** |  | 61.50 | ( | 10.00 | ) | **^c^** | 54.00 | ( | 10.00 | ) | ^a, b^ | **0.001*** | **< 0.001*** |
|  | 67.00 | ± | 10.18 |  |  |  | 60.45 | ± | 10.04 |  |  |  |  | 62.86 | ± | 8.45 |  |  | 53.75 | ± | 9.65 |  |  |  |  |
| **CpG 19** | 30.00 | ( | 26.00 | ) |  |  | 33.00 | ( | 27.00 | ) | 0.476 | 0.556 |  | 25.00 | ( | 25.00 | ) |  | 37.50 | ( | 22.00 | ) |  | 0.328 | 0.257 |
|  | 27.58 | ± | 18.57 |  |  |  | 31.22 | ± | 21.51 |  |  |  |  | 23.50 | ± | 17.23 |  |  | 32.94 | ± | 21.75 |  |  |  |  |
| **CpG 20** | 70.50 | ( | 20.00 | ) |  |  | 69.00 | ( | 11.00 | ) | 0.991 | 0.918 |  | 69.00 | ( | 11.00 | ) |  | 68.00 | ( | 17.00 | ) |  | 0.604 | 0.137* |
|  | 67.67 | ± | 10.78 |  |  |  | 70.86 | ± | 11.13 |  |  |  |  | 67.60 | ± | 8.05 |  |  | 74.70 | ± | 14.57 |  |  |  |  |
| **CpG 21** | 80.00 | ( | 33.00 | ) |  |  | 62.00 | ( | 24.00 | ) | **0.023*** | 0.184 |  | 61.00 | ( | 26.00 | ) |  | 64.00 | ( | 12.00 | ) |  | 0.062* | 0.142 |
|  | 78.36 | ± | 17.80 |  |  |  | 65.26 | ± | 19.95 |  |  |  |  | 67.67 | ± | 16.01 |  |  | 61.22 | ± | 18.75 |  |  |  |  |
| **CpG 24** | 70.50 | ( | 20.00 | ) |  |  | 69.00 | ( | 11.00 | ) | 0.991 | 0.918 |  | 69.00 | ( | 11.00 | ) |  | 68.00 | ( | 17.00 | ) |  | 0.604 | 0.137* |
|  | 67.67 | ± | 10.78 |  |  |  | 70.86 | ± | 11.13 |  |  |  |  | 67.60 | ± | 8.05 |  |  | 74.70 | ± | 14.57 |  |  |  |  |
| **CpG 25.26.27** | 64.00 | ( | 15.00 | ) |  |  | 61.00 | ( | 14.00 | ) | 0.202 | 0.474 |  | 60.50 | ( | 11.00 | ) |  | 60.00 | ( | 17.00 | ) |  | 0.351 | 0.752 |
|  | 60.00 | ± | 16.67 |  |  |  | 59.49 | ± | 9.86 |  |  |  |  | 59.45 | ± | 8.61 |  |  | 58.50 | ± | 11.41 |  |  |  |  |
| **CpG 28** | 68.00 | ( | 11.00 | ) | **^c^** |  | 60.00 | ( | 12.00 | ) | **0.001*** | **0.001*** |  | 63.00 | ( | 9.00 | ) | **^c^** | 56.50 | ( | 10.00 | ) | **^a, b^** | **< 0.001*** | **< 0.001*** |
|  | 67.78 | ± | 8.21 |  |  |  | 61.04 | ± | 10.06 |  |  |  |  | 64.32 | ± | 7.83 |  |  | 55.56 | ± | 8.76 |  |  |  |  |
| **CpG 29** | 100.00 | ( | 44.00 | ) | **^c^** |  | 57.00 | ( | 79.00 | ) | **0.017*** | **0.03*** |  | 86.00 | ( | 98.00 | ) |  | 35.00 | ( | 42.00 | ) | **^a^** | **0.01*** | **0.008*** |
|  | 75.32 | ± | 35.08 |  |  |  | 55.31 | ± | 39.13 |  |  |  |  | 59.88 | ± | 44.29 |  |  | 34.91 | ± | 25.56 |  |  |  |  |

Data are reported as median and interquartile range (IQ) and mean ± SD.

KW test: nonparametric comparison by the Kruskal–Wallis test. Pairwise comparisons, adjusted for multiple comparisons, were performed by the Dunn-Bonferroni test (comparisons with p < 0.05 are marked by the associated superscripts).

GLM test: group comparison by generalized linear model analysis. The models included the effects of age and sex as covariates. Bold text indicates significant P values (≤ 0.05). The asterisk (*) indicates significant difference after the Benjamini-Hochberg correction procedure at a FDR = 0.25.

*Table S6*. Correlation analysis between *SOCS3* CpG methylation and *SOCS3* expression and PAR.

|  | **Expression** | | **PAR** | |
| --- | --- | --- | --- | --- |
|  | **r coeff.** | ***P*** | **r coeff.** | ***P*** |
| **Expression** | - | - | 0.60 | **< 0.001*** |
| **CpG 5** | 0.12 | 0.514 | 0.06 | 0.691 |
| **CpG 6** | 0.27 | 0.252 | 0.25 | 0.159 |
| **CpG 8** | -0.23 | 0.217 | -0.16 | 0.269 |
| **CpG 9** | -0.25 | 0.176 | 0.11 | 0.446 |
| **CpG 10** | -0.24 | 0.186 | -0.17 | 0.242 |
| **CpG 11.12** | -0.51 | **0.004*** | -0.34 | **0.017*** |
| **CpG 13** | -0.36 | **0.048** | -0.52 | **< 0.001*** |
| **CpG 14** | -0.16 | 0.389 | -0.16 | 0.265 |
| **CpG 15.16** | -0.12 | 0.531 | -0.31 | **0.026*** |
| **CpG 17.18** | -0.25 | 0.185 | -0.29 | **0.042*** |
| **CpG 19** | 0.23 | 0.218 | 0.12 | 0.422 |
| **CpG 20** | 0.27 | 0.252 | 0.25 | 0.159 |
| **CpG 21** | -0.10 | 0.658 | -0.22 | 0.188 |
| **CpG 24** | 0.27 | 0.252 | 0.25 | 0.159 |
| **CpG 25.26.27** | -0.21 | 0.262 | -0.05 | 0.720 |
| **CpG 28** | -0.38 | **0.034** | -0.33 | **0.018*** |
| **CpG 29** | -0.40 | 0.082 | -0.20 | 0.231 |

N = 61 T2DM subjects. Bold text indicates significant P values (≤ 0.05). The asterisk (*) indicates significant correlation after the Benjamini-Hochberg correction procedure at a FDR = 0.25.
